# Efficiently Capturing Mitochondria-Targeted Constituents with Hepatoprotective Activity from Medicinal Herbs

**DOI:** 10.1155/2019/4353791

**Published:** 2019-04-09

**Authors:** Xing-Xin Yang, Li Liang, Xin Liu, Feng-Jiao Li, Jin-Cai Dong, Jian-Kang Mu, Lin-Xi Zeng, Qian Bi, Wen Gu, Jie Yu

**Affiliations:** ^1^College of Pharmaceutical Science, Yunnan University of Traditional Chinese Medicine, 1076 Yuhua Road, Kunming, 650500 Yunnan Province, China; ^2^Kunming Key Laboratory for Metabolic Diseases Prevention and Treatment by Chinese Medicine, 1076 Yuhua Road, Kunming, 650500 Yunnan Province, China; ^3^Beijing Entry-Exit Inspection and Quarantine Bureau, 6 Tianshuiyuan Street, Beijing 100026, China

## Abstract

Targeting mitochondria as a hepatic-protective strategy has gained attention, because of their important roles in energy production, adjustment of apoptosis, and generation of reactive oxygen species. To promote the discovery of natural mitochondria-targeted hepatic-protectants, we established a hepatocellular mitochondria-based capturing method by coupling affinity ultrafiltration with liquid chromatography/mass spectrometry (LC/MS), which is suitable for identifying mitochondrial ligands from medicinal herbs (MHs). After evaluating the feasibility of the method, it was applied for capturing mitochondria-targeting constituents from Peucedani Radix extract. A total of 10 active compounds were identified by LC/MS, all of which were newly identified mitochondrial ligands. The mitochondria-remedying activity of 4 of the 10 hits was confirmed by pharmacological tests *in vitro*. Additionally, the hepatic-protective abilities of 4 hits were verified in both carbon tetrachloride-damaged liver L02 cells and mice. These results indicated that the method could be used for identifying hepatic mitochondria-targeting constituents in MHs, which might be beneficial for hepatic-protective development.

## 1. Introduction

The liver is a complex organ that is involved in a myriad of essential life processes, including drug metabolism, protein production, and glucose storage. The liver is one of the most vulnerable organs of the body and can easily be damaged by a wide range of risk factors, such as viruses, cholestasis, steatosis, alcohol, and drugs [1]. Upon damage of hepatocytes, a series of changes will occur, including changes in lipid peroxidation and the release of cytokines, which aggravates liver fibrosis and may eventually lead to liver cirrhosis and carcinoma [2]. Therefore, protecting hepatocytes is of vital importance for curing a variety of acute and chronic liver diseases.

Mitochondria play a central role in complex processes, such as generation of energy and reactive oxygen species (ROS), maintaining calcium homeostasis, and adjusting apoptosis and lipid metabolism [3]. An increasing number of studies have indicated that mitochondrial dysfunction has become an important mechanism of liver injury and its aggravation, in which the following mechanisms may play a role [4–6]: (1) inhibition of mitochondrial *β*-oxidation and respiratory chain function, leading to electron transfer barriers that interfere with ATP synthesis, (2) opening of the mitochondrial membrane permeability transition (mPTP), which leads to collapse of the mitochondrial membrane potential (∆Ψm) and termination of ATP production, (3) destruction of the antioxidant defense system induced by production of a large number of ROS, resulting in oxidative damage and additional degradation of mtDNA, and (4) destruction of mitochondrial protein synthesis. Thus, strategies to prevent mitochondrial damage or to manipulate mitochondrial function in a clinically useful manner may provide effective therapies to treat hepatic injury [7].

Medicinal herbs (MHs) have been widely used to prevent liver diseases and have been reported as a highly important source for the development of promising hepatic-protectants. Several known mitochondria-targeted hepatic-protectants originated from natural constituents, such as salvianolate [2], daidzin (DZ) [8], and silybin (SB) [9]. A rapidly growing body of evidence has suggested that various MHs perform their hepatic-protective efficacy through regulating mitochondrial function [10, 11]. However, the specific bioactive constituents that are responsible for the therapeutic effects have not yet been identified. This severely hinders natural hepatic-protective development from MHs and limits further understanding of hepatic-protective mechanisms of MHs. Thus, screening of bioactive constituents within MHs with excellent protective effects against hepatocyte injury that act on mitochondria is of utmost importance.

The conventional approach for identifying mitochondria-targeted hepatic-protectants from MHs included extraction and isolation of MH constituents, followed by pharmacological testing of the purified chemicals. However, this was not successful for the direct search of hepatic-protectants from mixtures in addition to being labor-intensive and time- and sample-consuming [12]. In recent studies, high-throughput screening [13] and high-content screening methods [14] have been used for the rapid identification of mitochondrial ligands. These, however, were inefficient for direct capturing of multiple ligands from complex matrixes, because they were specifically designed to evaluate pure chemicals. In our previous study, a myocardial mitochondria-based screening method was developed for the identification of mitochondrial-associated ligands from MH extracts by combining centrifugal ultrafiltration (CU) with liquid chromatography/mass spectrometry (LC/MS), which resulted in many bioactive compounds [15, 16]. These results suggested that combining the CU technique and the LC/MS method would be a successful approach that may be applied for screening myocardial mitochondrial ligands from complex samples. Previous studies have reported numerous notable differences in the size, shape, number, and ultrastructure of mitochondria derived from different biological tissues. Moreover, to coordinate the different physiological needs of different tissues in an organism, these tissues were required to possess a specific gene expression system, which resulted in specific mitochondrial proteomes from different tissues [17]. For example, Mootha et al. demonstrated the abundant presence of different and specific mitochondrial proteins among the heart, liver, kidney, and brain, whereas only 50% of these mitochondrial proteins were shared [18].

**Figure 1:**
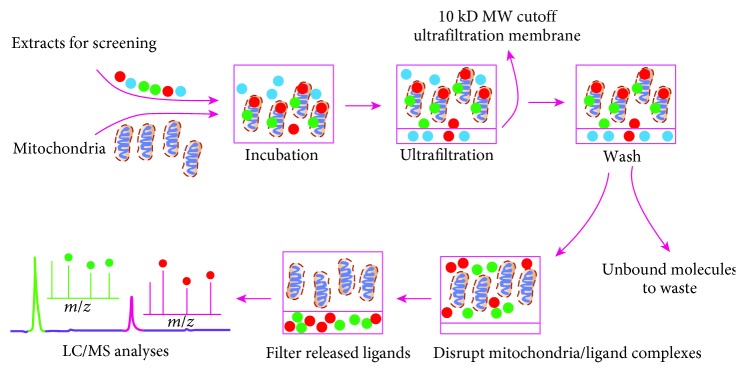
Overview of the analytical procedure of the search for mitochondria-targeted constituents from complex samples by CM-HMC.

To be compatible with the specific hunting of mitochondria-targeted hepatic-protectants from MHs used to against hepatic injury such as Peucedani Radix (PR), in the present study, we initially developed an efficient capturing method based on hepatic mitochondria (HM) by combining CU with LC/MS. In this method (Figure 1), named the capturing method for hepatic mitochondria-targeted compounds (CM-HMC), bioactive substances that selectively bound to HM were isolated by CU, after which the isolated fractions were injected into a LC/MS system for isolation and identification. Finally, the hepatic-protective activity of several hit compounds was confirmed *in vitro* and *in vivo* by pharmacological tests. The screening results are beneficial for the development of natural hepatic-protectants from MHs and in-depth understanding of hepatic-protective mechanisms of MHs. CM-HMC showed to be an efficient proposal for efficient screening of HM-targeted compounds from complex mixtures.

## 2. Material and Methods

### 2.1. Chemicals, Reagents, and Materials

SB, DZ, glucuronolactone (GL), praeruptorin A (**P8**), praeruptorin B (**P12**), praeruptorin D (**P13**), and praeruptorin E (**P15**) were purchased from Chengdu Pufeide Biological Technology Co. Ltd. (Chengdu, China). Cyclosporin A (CsA) was obtained from J&K Scientific Ltd. (Beijing, China). Amoxicillin (AC) and rhodamine 123 (Rh123) were provided by Dalian Meilun Biotechnology Co. Ltd. (Dalian, China). Pioglitazone hydrochloride (PH) was obtained from Ark Pharm Inc. (Chicago, IL, USA). Mitochondria Separation Kit which has been widely applied for separating intact and pure mitochondria from rat tissues such as liver and heat [19] was provided by GenMed Scientifics Inc. (Arlington, MA, USA). Bicinchoninic Acid (BCA) Protein Determination Kit was obtained from Nanjing Built Biological Engineering Research Institute (Nanjing, China). CCCP (10 mM) was purchased from Sigma-Aldrich (Saint Louis, MO, USA). HPLC-grade formic acid, methanol, and acetonitrile were supplied by Merck (Darmstadt, Germany). High-purity deionized water was purified by a Milli-Q System (Millipore, Bedford, MA, USA). All other reagents were of analytical grade or higher. Peucedani Radix (PR, purchase date June 16, 2016) was purchased from the Lvsheng Business Department of Medicinal Materials (Kunming, China). Samples were authenticated by Professor Jie Yu, and voucher specimen of PR (No. 8426) was deposited in the Key Laboratory of Preventing Metabolic Diseases of Traditional Chinese Medicine, Yunnan University of Traditional Chinese Medicine (Kunming, China).

### 2.2. Experimental Animals

Healthy male Sprague-Dawley rats (200 ± 50 g) and male Kunming mice (18-22 g) were obtained from Liaoning Changsheng Biotechnology Co. Ltd. (Liaoning, China). Throughout experiments, animals had access to standard chow diet and tap water *ad libitum* and were housed at a constant temperature and humidity under 12 h day/12 h night cycles. All procedures involving animals complied with the Guide for the Care and Use of Laboratory Animals as published by the US National Institutes of Health and were approved by the Institutional Ethical Committee on Animal Care and Experimentations of Yunnan University of Traditional Chinese Medicine (R-0620160026). Efforts were made to minimize suffering and the number of animals used.

### 2.3. Preparation of Analytical Solutions

Standard stock solutions (2 mg/mL) of SB, DZ, AC, and GL were prepared by dissolving the respective working standard substance in dimethyl sulfoxide (DMSO). Mixed standard solutions containing 2 mg/mL of SB, DZ, AC, and GL each were prepared in DMSO. The PR working solution (495 mg/mL) was prepared by dissolving freeze-dried powder of the PR extract (the preparation procedure is described in the Supplementary Information) in DMSO. For *in vitro* pharmacological trials, analytical solutions of standard substances derived from PR extract (**P8**, **P12**, **P13**, and **P15**) and used as pharmacological experimental tools (SB, CsA, and PH) were prepared in DMSO and diluted with physiological saline to the final concentrations. For *in vivo* pharmacological experiments, test solutions of **P12** were prepared in Tween-80 and diluted with physiological saline to the final concentrations. Test solutions of SB were directly prepared in physiological saline. All solutions were stored at 4°C in the dark.

### 2.4. Preparation of a HM Suspension

HM were prepared from rat liver using Mitochondria Isolation Kit (which employs organelle release followed by differential centrifugation) in adherence to the manufacturer's recommendations. All procedures were performed on ice or at 4°C in a cold room. Briefly, a Sprague-Dawley rat was sacrificed by decapitation, and then the liver was quickly excised and placed in ice-cold Reagent A. After trimming and washing twice with Reagent A to remove any remaining blood, the liver was minced into 1 mm^3^ pieces with scissors, followed by homogenization with a Dounce glass homogenizer (Kimble/Kontes, Vineland, NJ, USA) using Working Solution (3.2 mL Reagent B plus 0.8 mL Reagent C and 16 *μ*L Reagent F). Subsequently, the tissue homogenate was centrifuged at 1,000 × *g* for 10 min. The supernatant was collected and centrifuged at 10,000 × *g* for 10 min. The pellet, which contained mitochondria, was resuspended in Reagent E to produce a HM suspension (1.0 g/L). The mitochondrial protein concentration was measured by BCA assay. In addition, a portion of the HM suspension was persistently heated for 2 h in boiling water to produce denatured mitochondria that had lost their primary biological function (Figure S1).

### 2.5. Integrity and Purity Evaluation of HM

The structural intactness, biological functions, and purity of prepared HM fractions were evaluated through transmission electron microscopy and ∆Ψm determination followed by Rh123 fluorescence quenching and a commercial Mitochondrial Fraction Purity Marker Enzyme Assay Kit (Mito-Purity Enzymatic Determination Kit, GenMed Scientifics Inc., Arlington, MA, USA), respectively. Experimental procedures are described in the Supplementary Information.

### 2.6. Screening Procedure of CM-HMC

The screening process of SM-HBC was similar to the method reported previously [15]. Analytical solutions (5 *μ*L) including standard solutions, a mixed standard solution, and a PR working solution were incubated with the mitochondria suspension (200 *μ*L) for 90 min at 37°C to allow complete binding between the solutes and mitochondria. The mixture was then filtered through a 0.5 mL centrifugal filter (Microcon YM-10, Millipore Co., Bedford, MA, USA) with a 10 kDa molecular weight cut-off ultrafiltration membrane by centrifugation at 14,000 × *g* for 25 min at 4°C. The mitochondria/ligand complexes trapped in the membrane were washed three times with 200 *μ*L of ammonium acetate buffer (50 mM, pH 7.5) by centrifugation at 14,000 × *g* for 25 min at 4°C to eliminate nonspecifically bound solutes. Next, the bound ligands were released from the mitochondria by ultrasonication in 80% aqueous methanol (400 *μ*L) for 20 min followed by centrifugation at 14,000 × *g* for 25 min at room temperature. The ultrafiltrate-containing ligands were then dried under a nitrogen stream. After reconstitution with 100 *μ*L of 80% aqueous methanol, the sample solutions were analyzed by LC/MS. Control tests to determine nonspecific binding were undertaken in a similar fashion using denatured mitochondria. LC/MS peaks that significantly enhanced in area in the experiments compared to the control containing denatured mitochondria (when the difference of peak area between experiment and control (Δ*P*) was >20%) indicated specific binding and were deemed as ligands bound to mitochondria. All screening trials were independently performed at least in triplicate and analyzed in duplicate. The Δ*P* values were calculated as follows:

(1) $$\begin{array}{c} \Delta P=\left(P_{\text{e}}–P_{\text{c}}\right)/P_{\text{e}}\times 100, \end{array}$$

where *P*_e_ and *P*_c_ represented the peak areas in the experiment and control, respectively.

### 2.7. Analytical Conditions of LC/MS

LC/MS analyses were performed on an UHPLC Dionex Ultimate 3000 system coupled to a Thermo Scientific Q-Exactive™ hybrid quadrupole-orbitrap mass spectrometer with a heated-electrospray ionization probe (Thermo Fisher Scientific, San Jose, CA, USA). The UHPLC system consisted of a quaternary pump, an autosampler with a temperature control function, a column box, and a photodiode array (PDA) detector. For all analyzed samples, the UHPLC-PDA conditions are listed in Table S1. The HESI-MS^n^ parameters for all samples were as follows: (1) flow rate: 0.2 mL/min (split from HPLC effluent); (2) detection mode: positive and negative ion; (3) heat block and curved desolvation line temperature: 250°C; nebulizing nitrogen gas flow: 1.5 L/min; interface voltage: (+) 3.5 kV, (-) -2.8 kV; (4) mass range: MS, *m*/*z* 100-1000; MS^2^ and MS^3^, *m*/*z* 50-1000; (5) use dynamic exclusion, dynamic exclusion time: 10 s; and (6) workstation: Xcalibur 3.0.63 for liquid chromatography combined with data processing, molecular prediction, and precise molecular weight calculations.

### 2.8. Determination of mPTP Opening, ∆Ψm, and ATPase Activity in Isolated HM

Opening of the mPTP, which was measured by Ca^2+^-induced swelling of isolated HM, causes mitochondrial swelling that reduces the absorbance at 520 nm (A_520_). Mitochondrial swelling was determined following a method reported previously [15]. In brief, isolated HM were resuspended in swelling buffer (120 mM KCl, 20 mM MOPS, 10 mM Tris-HCl, and 5 mM KH_2_PO_4_, pH 7.4) to a final concentration of 0.25 g/L, incubated with the assayed compounds for 3 min at room temperature, followed by addition of 250 *μ*M CaCl_2_ to induce mPTP opening for 15 min. Then, A_520_ was determined using a UV–VIS spectrophotometer (AOE Instruments (Shanghai) Co. Ltd., Shanghai, China). Experiments were performed in triplicate.

∆Ψm was determined by Rh123 fluorescence quenching [20]. At high ΔΨm levels, most Rh123 is concentrated in the mitochondrial matrix and is quenched. At lower ΔΨm levels, however, Rh123 is released, thereby inducing dequenching resulting in an increase in Rh123 fluorescence. In brief, isolated HM were resuspended in measurement medium (0.25 M sucrose, 5 mM MgCl_2_, 10 mM KCl, 5 mM KH_2_PO_4_, 10 mM HEPES, and 10 mM succinate, pH 7.4) to a final concentration of 1 g/L and incubated with the tested compounds and Rh123 (5 *μ*L; 1 g/L) for 30 min at 37°C, followed by addition of 880 *μ*M PH to induce the release of Rh123 for 15 min. Changes of ΔΨm-dependent quenching of Rh123 fluorescence were measured in triplicate by an Infinite M200 PRO Multi-functional Microplate Reader (Dongsheng Innovation Biotechnology Co. Ltd., Beijing, China) set to wavelengths of 484 nm excitation and 534 nm emission.

ATPase activity was measured by PH-induced injury of isolated HM. In brief, isolated HM were resuspended in a measurement medium to a final concentration of 1 g/L, incubated with the tested compounds for 20 min at 37°C, followed by addition of 880 *μ*M PH to induce a decrease in ATPase activity for 20 min. Changes in Na^+^-K^+^-ATPase and Ca^2+^-Mg^2+^-ATPase activity were determined on a SpectraMax Plus 384 Microplate Reader (Molecular Devices, Sunnyvale, CA, USA) using an ATPase Determination Kit (Nanjing Built Biological Engineering Research Institute, Nanjing, China) according to the manufacturer's instructions.

### 2.9. In Vitro Hepatoprotective Activity Assay

L02 cells (1 × 10^5^ cells per well) were plated into each well of a 96-well plate and incubated in Dulbecco's modified Eagle's medium (DMEM) supplemented with 10% fetal bovine serum (FBS) at 37°C in a humidified atmosphere of 5% CO_2_. After cells were attached to the plates, they were treated with the tested compounds at different concentrations and cultured for 18 h at 37°C. Then, 10 *μ*M of CCl_4_ was added to induce hepatocellular injury for 6 h, and cell viability (CCK-8 assay), level of MDA, and enzyme activities of ALT, AST, and SOD were measured using commercially available diagnostic kits (Nanjing Built Biological Engineering Research Institute, Nanjing, China) in accordance with the manufacturer's instructions using a SpectraMax Plus 384 Microplate Reader (Molecular Devices, Sunnyvale, CA, USA).

### 2.10. In Vivo Hepatoprotective Activity Assay

The mouse model of CCl_4_-induced liver damage was used for assaying *in vivo* hepatoprotective effects of the hit compounds [21]. Mice were randomly allocated into six groups (10 mice per group), including normal, model, 8 mg/kg **P12**, 16 mg/kg **P12**, 32 mg/kg **P12**, and 16 mg/kg SB groups. Mice in the normal and model groups were administered 10 mL/kg physiological saline each day for 7 consecutive days. Mice in the three **P12** groups received 8, 16, and 32 mg/kg **P12** solution at 10 mL/kg per day by intragastric administration (i.g.) for 7 consecutive days. The SB (16 mg/kg) group was used as a positive control, and these mice were administered 10 mL/kg SB solution per day for 7 consecutive days. At day 7, mice in all groups, except for mice in the normal group, were intraperitoneally (i.p.) injected with 0.1% CCl_4_ in olive oil (10 mL/kg body weight) to induce hepatic injury. After 16 h, mice were sacrificed by decapitation. Blood was collected to determine ALT and AST activities, and liver tissues were collected. A portion of liver samples were immediately used for measuring ∆Ψm and mPTP, while the remaining samples were preserved at -80°C until use. Biological assays included histopathological examination and determination of antioxidant indexes in liver tissues (SOD, MDA, and GSH) and mitochondria-involved indexes (MDA, Na^+^-K^+^-ATPase, Ca^2+^-Mg^2+^-ATPase, ∆Ψm, and mPTP). All the measurement procedures of the indexes are presented in the Supplementary Information.

### 2.11. Statistical Analysis

Data are expressed as mean ± SD. Statistical analysis was performed using SPSS software (13.0 for Windows; SPSS, Chicago, IL, USA). Differences between two groups were analyzed by two-tailed Student's *t*-test, while differences between three or more groups were analyzed by one-way analysis of variance (ANOVA, Dunnett's method). *P* < 0.05 (two-tailed) was considered statistically significant.

## 3. Results and Discussion

### 3.1. Acquisition of Intact and Pure HM

The key to establishing a mitochondria-based screening method was to obtain plenty of intact and pure HM. Firstly, the mitochondria-specific dye Janus green B was used along with neutral red (which stains lysosomes in red and can stain the Golgi apparatus) [22] for the initial observation of HM. The mitochondrial fraction was successfully stained with Janus green B, whereas the (excluded) nonmitochondrial fraction was stained with neutral red (Figure S2), indicating that bioactive HM was successfully isolated and enriched from hepatic tissues using the mitochondria isolation kit, which employs organelle release followed by differential centrifugation.

**Figure 2:**
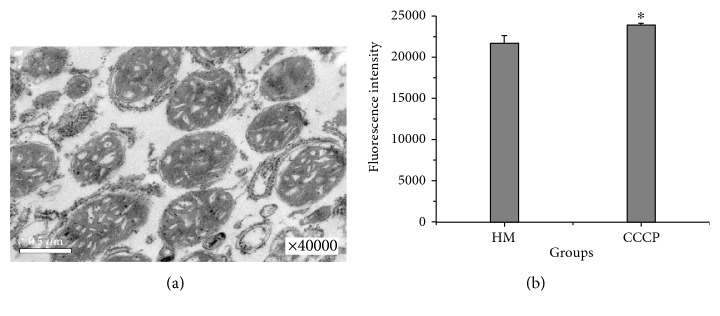
Evaluation of the structural integrity and bio-functions of HM. (a) Transmission electron micrograph displaying typical mitochondrial morphology (×40,000). (b) Changes in the ΔΨm of HM after CCCP treatment. ΔΨm was determined as the difference in Rh123 uptake by HM and CCCP-treated HM and expressed in units of fluorescence intensity. HM: hepatic mitochondria. Data were obtained from 3 independent experiments and expressed as the mean ± SD. ^∗^ *P* < 0.05 compared with HM.

The structural integrity, biological functions, and purity of the prepared HM fractions were evaluated. The representative electron micrographs of isolated HM shown in Figure 2(a) revealed that the mitochondrial cristae and membrane were intact and continuous, which indicated the high structural integrity of the HM. The satisfactory results in which the fluorescence intensity of the HM fraction was remarkably augmented after treatment with CCCP (a mitochondrial uncoupler that can strongly break the ΔΨm [15] (Figure 2(b)) suggested collapse of the ΔΨm of the HM. This indicated that enriched HM still possessed their biological functions. The results shown in Figure 2(a) presented substantially fewer impurities in the HM, indicating a high level of purity. Lysosomes are not easy to be removed during the procedure of mitochondrial enrichment and are a possible contaminant in the HM fraction. Therefore, the lysosome content in the HM fraction relative to mitochondria was determined by assaying their corresponding marker enzymes and was performed following the manufacturer's instructions [23]. The results showed that the mitochondria-marker enzyme (succinate dehydrogenase) was at least 20 times higher compared to that of the lysosome-marker enzyme (acid phosphatase) in purified mitochondria, suggesting a high level of purity.

It is highly possible that there are still some trace impurities in the HM. However, these trace amounts will not affect the screening of active constituents by CM-HMC for two reasons: (1) almost all active substances may be bound to abundant HM, but not to possible trace impurities, and (2) despite the fact that several chemicals in MHs are potentially bound to trace impurities, they may not be detected by LC/MS analysis, due to the significantly low contents of the bound chemicals.

### 3.2. Feasibility of CM-HMC

**Figure 3:**
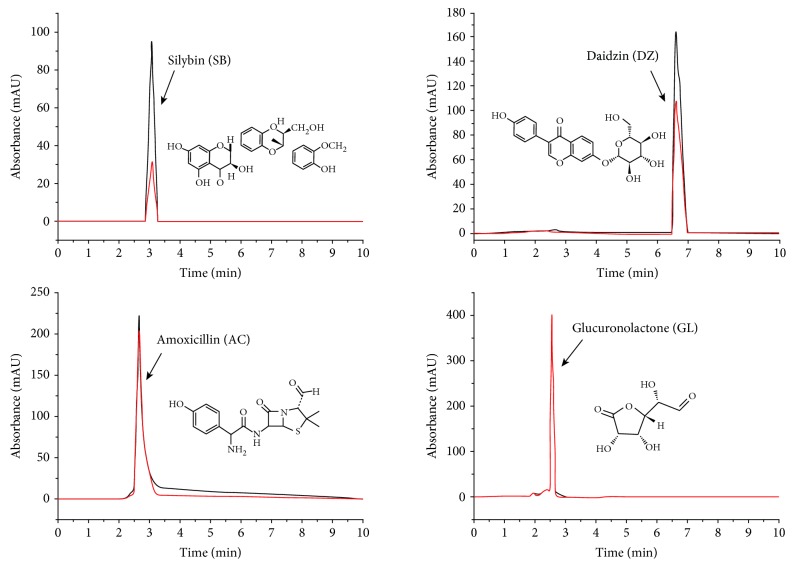
Analysis of four standard solutions by CM-HMC. HPLC chromatograms of ultrafiltrates comprised of standards released from active HM (black line) or denatured HM (red line), which served as a control, are presented. Enhancement of the peak area of the standards compared with the control indicates specific binding with HM.

Standard solutions including SB, DZ, AC, and GL, and a standard solution containing of the four standards, were prepared for evaluating the feasibility of the novel CM-HMC method, using denatured HM as a control (red line). The standard solutions were separately analyzed by CM-HMC, and the obtained HPLC chromatograms are presented in Figure 3. The SB and DZ peaks showed remarkable enhancements in the peak area compared with the control, which contained denatured HM (Δ*P* values shown in Table 1 were﹥20%), demonstrating specific binding with active HM. However, the peak areas of AC and GL were nearly equal to that of the control (Δ*P* < 20%, shown in Table 1). MS data (Table 1) verified that the peaks corresponded to SB, DZ, AC, and GL. Hence, two of the standards (SB and DZ) that specifically bound with HM were identified by CM-HMC.

**Figure 4:**
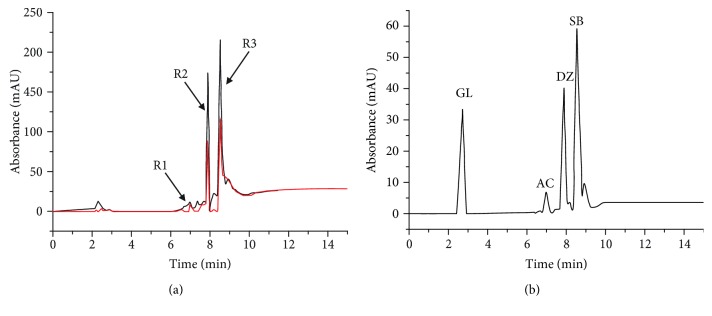
Determination of a mixed standard solution using CM-HMC. (a) HPLC chromatograms of a mixed standard solution are shown for ultrafiltrates derived from active HM (black line) and denatured HM (red line), which served as a control. Peaks R2 and R3 exhibited remarkable area enhancement compared with the control. However, the area of the R1 peak was equal to that of the control. (b) HPLC chromatogram of a mixed standard solution, which was directly assayed by LC/MS. GL: glucuronide; AC: amoxicillin; DZ: daidzin; and SB: silybin.

Additionally, the mixed standard solution was assayed by CM-HMC and directly analyzed by LC/MS. The HPLC chromatograms are displayed in Figures 4(a) and 4(b), respectively. When compared with the control, the prominent enhancement of peaks R2 (Δ*P* = 36.4 ± 8.3%, *n* = 3) and R3 (Δ*P* = 57.3 ± 10.3%, *n* = 3) clearly indicated specific binding with HM, whereas the R1 peak was nearly identical to that of the control (Δ*P* = 4.8 ± 2.1%, *n* = 3). Comparing the retention times of the peaks (Figures 4(a) and 4(b)) indicated that the R1, R2, and R3 peaks corresponded to AC, DZ, and SB, respectively. GL could not be detected in the screening process. Thus, by using the screening novel method, DZ and SB were found to specifically bind with HM and were captured from the mixture solution.

These above-mentioned results suggested that SB and DZ specifically bound with HM with Δ*P* > 20%, whereas AC and GL did not (Δ*P* < 20%). Therefore, peaks with significant area enhancements (Δ*P* > 20%) were considered as active compounds with specific HM-binding activity. SB [9, 24] and DZ [25] inhibited the activities of the mitochondrial electron transfer chain and monoamine oxidase, respectively, thereby decreasing the levels of ROS. Hence, the interaction between the tested compounds and the targets in mitochondria may result in specific binding of SB and DZ to mitochondria. AC [26], a *β*-lactam antibiotic that inhibits the synthesis of bacterial cell walls, did not specifically bind to HM but selectively acted on penicillin-binding proteins abundant in bacteria. In addition, GL [27, 28], a hepatic antidote, did not specifically bind to HM. The lactone ring in GL opens under enzymatic catalysis and combines with hydroxyl, carboxyl, and mercapto of hepatic toxicants to form nontoxic or low-toxic compounds that are directly eliminated *in vitro*. Thus, in the established CM-HMC, active molecules (SB and DZ) that bind to certain target sites (such as monoamine oxidase) in HM were selectively recognized, whereas inactive impurities (AC and GL) were directly excluded by the CU. The structural characteristics of the corresponding fractions were conveniently profiled by the LC/MS approach. Therefore, CM-HMC had the capabilities of recognition, separation, and identification.

### 3.3. Effect of Screening Conditions

The validated CM-HMC was applied for capturing of mitochondrial ligands from the PR extract. The effect of several significant screening conditions of the method, such as HM concentration, sample concentration, and incubation time, were evaluated to achieve the highest screening performance. First, different HM concentrations (0.25, 0.50, and 1.0 g/L) were used to capture bioactive constituents from the PR extract. The number of active molecules captured remarkably increased with the increase in HM concentration (Figure S3), which corresponded with previously reported screening results of other MHs in which myocardial mitochondria were used as targets [15]. Thus, the screening sensitivity is determined by the HM concentration and a higher HM concentration will result in a higher sensitivity, which may result in more active molecules from MHs. Higher HM concentrations (>1.0 g/L) will block the ultrafiltration membrane; therefore, for the CM-HMC method, 1.0 g/L was chosen as the optimum HM concentration, which allowed for screening of the maximum number of active molecules from MHs.

Next, three concentrations of the PR extract (3.10, 6.19, and 12.38 g/L) were tested for screening of active compounds. The number of active compounds identified increased with an increase in sample concentration (Figure S4). A sample concentration of 12.38 g/L was found to be optimal for capturing of the PR extract. Thus, sample concentration affected the hunt for mitochondria-targeted compounds and is recommended to be optimized according to the chemical constituents of samples analyzed by CM-HMC.

Finally, the effect of incubation time (60, 90, and 120 min) for capturing of active molecules from the PR extract was evaluated. Our findings showed that the maximum number of active molecules was captured at an incubation time of 90 min (Figure S5). Thus, incubation time also affected the screening results. When a short incubation time is used, active molecules that loosely bind HM may be missed, whereas a long incubation time may lead to structural changes of the bound molecules.

### 3.4. Application to PR Extract

**Figure 5:**
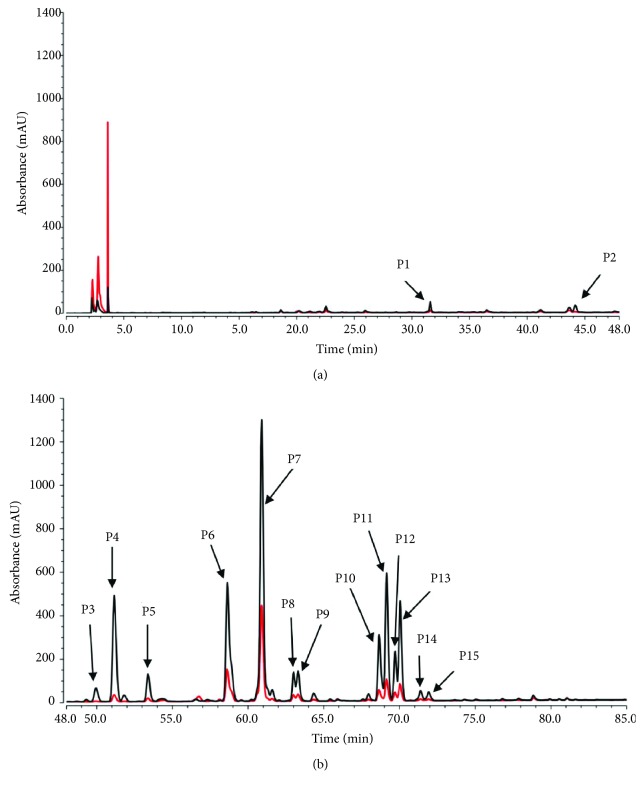
Screening of the PR extract for HM-targeted constituents by CM-HMC. Compared with the control, which was comprised of denatured HM (red line), HPLC chromatograms of screened PR extract (A and B; (a) 0–48 min; (b) 48–85 min) presented fifteen peaks (P1–P15) that were significantly enhanced due to specific binding with HM (black line).

**Figure 6:**
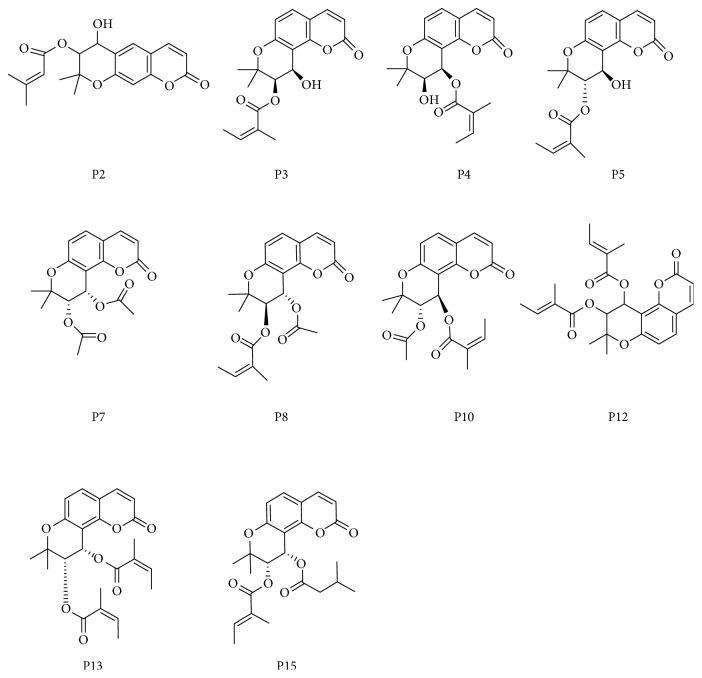
Chemical structures of the ten bioactive compounds that were identified from the PR extract.

CM-HMC was initially used to identify active constituents from the PR extract that bound with HM.  Figure 5 displays the chromatogram of the PR sample determined by CM-HMC, which reveals prominent enhancement in the peak area of 15 peaks (P1–P15) when compared with the control that contains denatured HM (Δ*P* > 20%, shown in Table 2). This indicated specific binding with active HM. After obtaining UV, MS, and MS*^n^* information from LC/MS (Table 2) and by comparing with previously reported data [29–33] and standards, the chemical structures of 10 peaks (Figure 6) were assigned to as coumarin. It is important to point out that several constituents with a low peak area in the PR extract (Figure 6) that showed area enhancement may have activity, but should not be marked as HM ligands, considering they were unrepeatedly captured by CM-HMC analysis. This may be attributed to the low abundance in the sample, low response to LC/MS, and/or weak binding with HM.

Moreover, these results further validated that CM-HMC could be used as an efficient alternative for capturing HM-targeted compounds from complex samples such as MHs. Active HM enriched from rat liver could recognize target molecules not only through affinity interactions between molecules and targets in HM environments but only through electrostatic interactions between them [15], which was beneficial for decreasing the interference from inactive impurities. The recognized fractions from MH extracts were rapidly isolated using the CU technique followed by LC/MS analyses.

It is clearly noted that CM-HMC could not be performed to distinguish between molecules that accumulate in the intermembrane space and the matrix compartment. Additionally, the accumulation of the compounds into mitochondria might occur via partitioning, depending on transporters, or the membrane potential. Due to the different structures and characters (such as molecular weight and polarity) of these hits, they might accumulate into HM by different ways, which merit further investigation.

### 3.5. Effects of Hit Compounds on mPTP Opening, ∆Ψm, and ATPase Activity in Isolated HM

**Figure 7:**
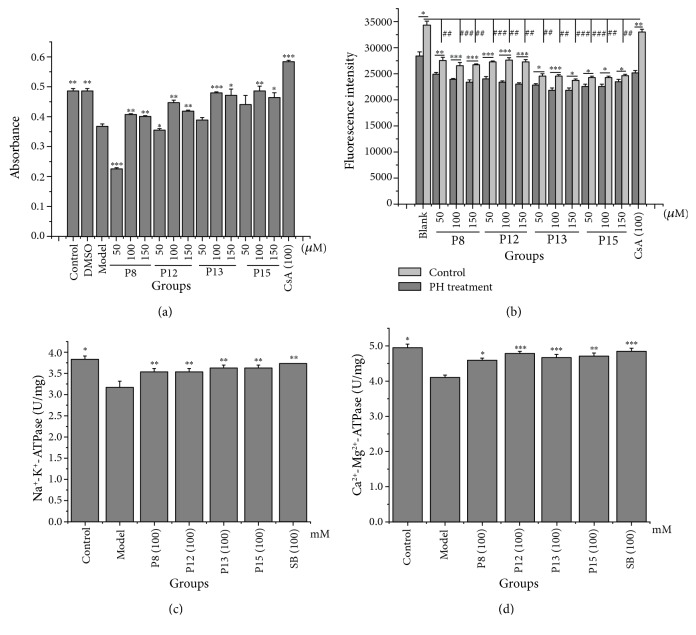
Effects of hits on the mPTP opening (a), ∆Ψm (b), Na^+^-K^+^-ATPase activity (c), and Ca^2+^-Mg^2+^-ATPase activity (d) in isolated HM. Data were obtained from 5 independent measurements and are expressed as mean ± SD. The statistical significance of differences between groups was assayed by one-way analysis of variance (ANOVA) using Dunnett's method. ^∗^ *P* < 0.05, ^∗∗^ *P* < 0.01, and ^∗∗∗^ *P* < 0.001 compared with the model control group (a, c, and d) or between the control and PH treatment (B), under identical conditions. ^##^ *P* < 0.01 and ^###^ *P* < 0.001 compared with the black group (PH treatment) measured under identical conditions. P8: praeruptorin A; P12: praeruptorin B; P13: praeruptorin D; P15: praeruptorin E; CsA: cyclosporin A; SB: silybin; PH: pioglitazone hydrochloride.

To confirm the mitochondria-binding abilities of these hit compounds and to evaluate their capabilities to regulate HM function, three mitochondrial indexes, which play a pivotal role in the maintenance of mitochondrial function, including mPTP opening, ∆Ψm, and ATPase activity, were analyzed. As shown in Figure 7(a), 250 *μ*M CaCl_2_ (which opens the mPTP [15]) induced a significant decrease in the absorbance (520 nm) of HM suspensions. This decrease was significantly inhibited by treatment with 10 *μ*M CsA, which specially prevented opening of the mPTP [15], suggesting that the decrease in absorbance was attributed to mPTP opening. As for CsA, the four tested compounds (**P8**, **P12**, **P13**, and **P15**) significantly inhibited the Ca^2+^-induced decrease in absorbance.

As shown in Figure 7(b), the fluorescence intensity of HM suspensions was significantly enhanced after treatment with PH, indicating a reduction in ∆Ψm of the HM. However, when the HM suspensions were pretreated for 30 min by the four tested compounds (**P8**, **P12**, **P13**, and **P15**), the enhancement of fluorescence intensity evoked by PH was significantly prevented. This suggested that these compounds inhibited the PH-induced decline of ∆Ψm of HM.

As shown in Figures 7(c) and 7(d), the Na^+^-K^+^-ATPase and Ca^2+^-Mg^2+^-ATPase activity of HM suspensions was significantly reduced after PH treatment. However, when HM suspensions were pretreated by **P8**, **P12**, **P13**, and **P15** for 20 min, the PH-induced decreases in ATPase activity were significantly inhibited, to a similar extent as the positive control (SB).

Together, these data indicated that the tested compounds were potential HM-targeted molecules that directly affected mitochondrial function, demonstrating that reliable results were obtained by CM-HMC analysis. In combination with the screening results supported by CM-HMC, the other 11 hits represented potential HM-targeted compounds that affected HM function, which merit further investigation. Additionally, it is unclear if these compounds mainly affect certain mitochondrial functions such as ATP synthesis, membrane potential, apoptosis, calcium homeostasis, and respiration, which also merit further investigation. The validation of certain main mitochondrial functions will be useful for dividing the hits into functional classes to define the potential application.

### 3.6. In Vivo and In Vitro Hepatoprotective Activities of Hit Compounds

**Figure 8:**
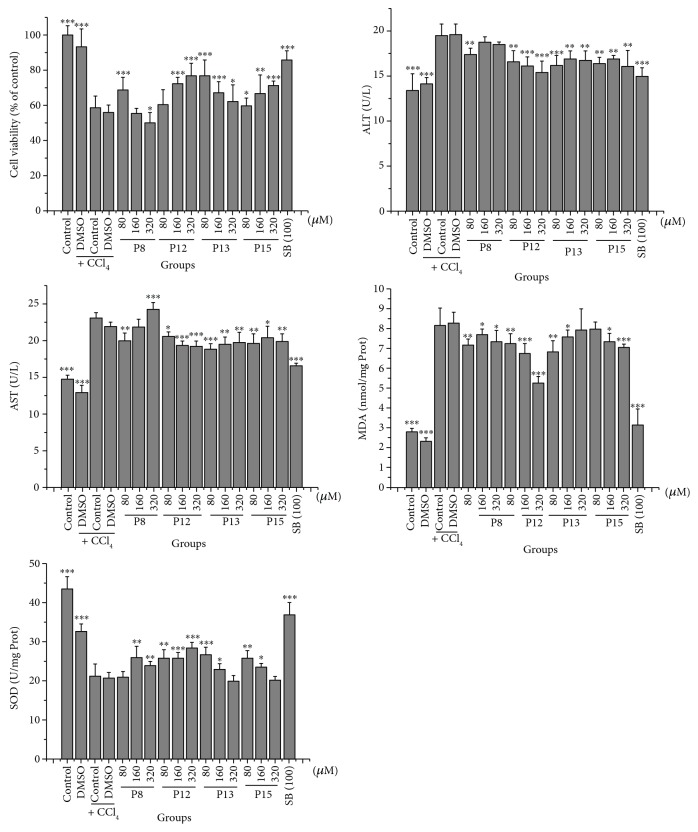
Effects of hit compounds on the hepatocytes of CCl_4_-induced injury. Data were obtained from 5 independent determinations and are expressed as mean ± SD. The statistical significance of differences between groups was evaluated by one-way analysis of variance (ANOVA) using Dunnett's method. ^∗^ *P* < 0.05, ^∗∗^ *P* < 0.01, and^∗∗∗^ *P* < 0.001 compared with the model control group under identical conditions. P8: praeruptorin A; P12: praeruptorin B; P13: praeruptorin D; P15: praeruptorin E; CsA: cyclosporin A; SB: silybin.

**Figure 9:**
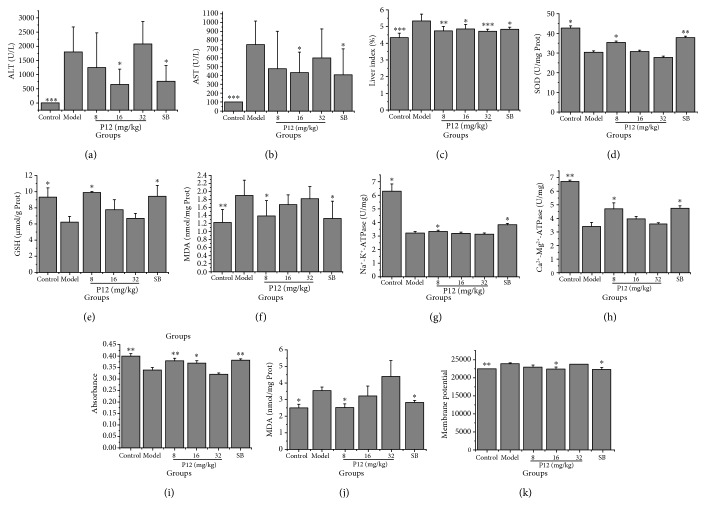
Effects of the hit compound on the mice of CCl_4_-induced injury. ALT and AST in serum (a and b), liver index, SOD, GSH, and MDA in liver tissue (c–f), and Na^+^-K^+^-ATPase, Ca^2+^-Mg^2+^-ATPase, mPTP, MDA, and ∆Ψm in HM (g–k) were measured. Data were obtained from 5 independent experiments and are expressed as mean ± SD. The statistical significance of differences between groups was evaluated by one-way analysis of variance (ANOVA) using Dunnett's method. ^∗^ *P* < 0.05, ^∗∗^ *P* < 0.01, and ^∗∗∗^ *P* < 0.001 compared with the model control group under identical conditions. P12: praeruptorin B; SB: silybin.

The hepatoprotective activities of the hit compounds were first assessed in a hepatocyte model of CCl_4_-induced injury. As demonstrated in Figure 8, CCl_4_ resulted in the significant decrease in cell viability and SOD and the notable increase in ALT, AST, and MDA, for hepatocytes (*P* < 0.001). These effects were significantly inverted by SB which can remedy mitochondrial dysfunction to protect hepatocytes [34]. Similar to SB, the four tested hits (**P8**, **P12**, **P13**, and **P15**) remarkably prevented CCl_4_-induced changes (*P* < 0.05). This may be attributed to the direct binding with HM that further stimulates CCl_4_-induced alterations and merits further investigation. The pharmacological effects of the tested hits appeared to have marginal effects in cultured cells, but they might show stronger activities on a pathologic animal model (e.g., **P12**, shown in Figure 9). Additionally, they can be served as the lead molecules that are modified to obtain the analogues with stronger activities. Whether the other 11 hit compounds also affected HM function to exert hepatocellular protection activities remains unclear.

**Figure 10:**
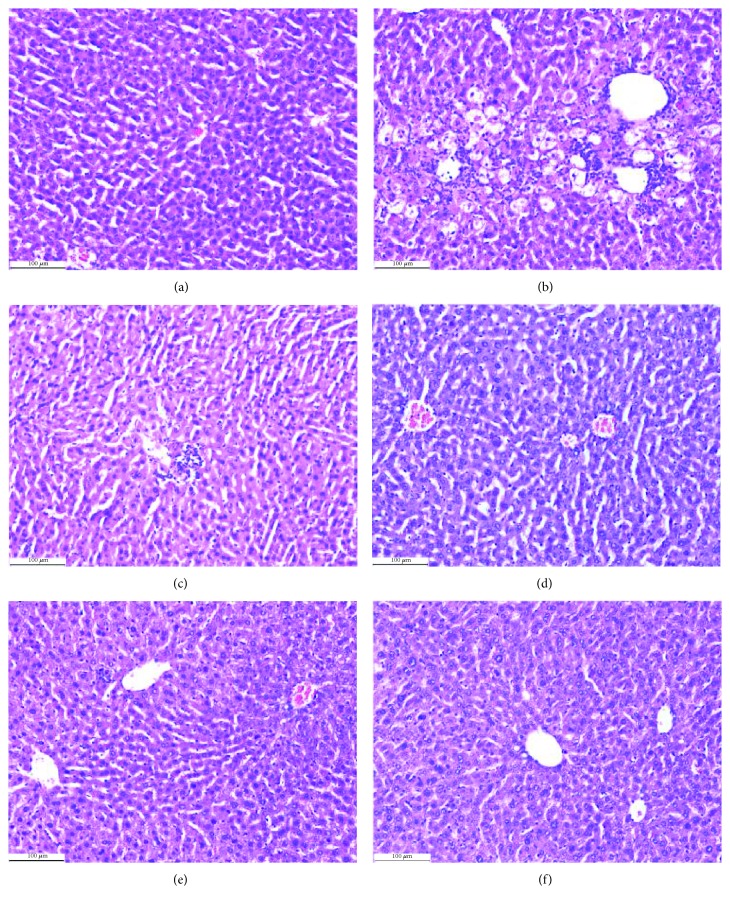
Effect of the hit compound on the hepatic histomorphology injury by CCl_4_. As shown in (a), normal liver displayed a typical hepatolobular architecture, comprised of a clear central vein with radiating cords of hepatocytes separated by sinusoids. Hepatic cells were polygonal in shape, with distinctive nuclei and a uniform cytoplasm. Few binucleated cells were present, and the cytoplasm was regularly distributed. As shown in (b), hepatic injuries induced by CCl_4_ in mice were demonstrated by marked vacuolization of hepatocytes, necrosis around the central vein, sinusoidal dilation and congestion, infiltration of cells, loss of cellular boundaries and ballooning degeneration, and loss of architecture and were significantly different from those observed in the normal control group. However, treatment with silybin (c) and P12 (praeruptorin B) at doses of 8, 16, and 32 mg/kg for 7 days are shown in (d), (e), and (f), respectively and significantly attenuated liver injuries as shown by the absence of focal or bridging necrosis or mild hepatitis. The CCl_4_-induced histopathological changes improved by treatment with P12, and the recovery was significant in the low-dose group (8 mg/kg), which was comparable to that of silybin.

A mouse model of CCl_4_-induced injury was used to confirm the *in vivo* hepatoprotective activity through regulating HM functions of the hit compound. As shown in Figures 9(a)–9(f) and Figure 10, CCl_4_ resulted in a notable elevation of the liver index, ALT, AST, and MDA, a significant reduction in SOD and GSH levels, as well as obvious injury in liver histomorphology, in mice. These effects were significantly inverted by test compound **P12**, which is similar as SB and can remedy mitochondrial dysfunction to exert hepatoprotective efficacy [9]. In addition, the effects of **P12** on HM (including MDA, ∆Ψm, mPTP opening, Na^+^-K^+^-ATPase, and Ca^2+^-Mg^2+^-ATPase activity) from CCl_4_-injury induced mice were assessed. As shown in Figures 9(g)–9(k), CCl_4_ treatment caused a notable reduction in Na^+^-K^+^-ATPase activity, Ga^2+^-Mg^2+^-ATPase activity, and absorbance at 520 nm (indicating mPTP opening), and the remarkable elevation in the content of MDA and fluorescence intensity (indicating the ∆Ψm decline), in HM of mice. These effects were significantly inverted by **P12**, such as SB. These results indicated that **P12** effectively inhibited CCl_4_-induced hepatic injury through remedying CCl_4_-induced HM dysfunction. This may be caused by eliminating oxidative stress products (MDA) and by improving the activity of antioxidant enzymes (SOD and GSH-PX) and energy metabolism obstacles. Nevertheless, whether the other 14 hit compounds can also regulate HM function to exert hepatoprotective activity *in vivo* has yet to be revealed.

The screening results from CM-HMC combined with the results of mitochondrial indexes in isolated HM; the above results suggested that all hit compounds may directly act on mitochondria to regulate HM function that further exhibit their hepatoprotective capabilities. Thus, our findings may be promising for the development of novel drugs from MHs and for elucidating the mechanism of action involved.

## 4. Conclusion

In this study, an efficient CM-HMC for capturing HM-targeted molecules from MHs, such as PR, was established. This method showed preeminent recognition, separation, and identification ability, with advantages such as a simple procedure, efficient usage of labor, and a saving of sample and time. Using CM-HMC, fifteen HM-targeted molecules were successfully captured from the PR extract, and the direct mitochondria-bound activity of four hit compounds in isolated HM was validated by evaluating mPTP opening, ∆Ψm, and ATPase activity. Furthermore, the hepatoprotective abilities of four hit compounds were identified by cell trials, and *in vivo* hepatoprotective activity through regulating HM function of one hit (**P12**) was further confirmed by animal tests. The results obtained will assist in the development of HM-targeted hepatic-protectants from MHs and in-depth interpretation of therapeutic principles of MHs. Thus, CM-HMC was validated to be a promising approach for efficiently capturing HM-targeted molecules from a mixture of samples.

## Figures and Tables

**Table 1:** LC/MS data for the four standard solutions.

Standard	*t*_R_ (min)	Δ*P* ^a^ (%, *n* = 3)	UV *λ*max (nm)	[M+H]^+^ m/z	ESI-MS*^n^* (+) *m*/*z* (abundance)	[M-H]^+^ *m*/*z*	ESI-MS*^n^*(-) *m*/*z* (abundance)
Silybin	3.087	56.3 ± 15.5	290	483.1282	—	481.1146	MS^2^ (481):125 (100), 301 (44), 179 (27), 453 (14), 463 (10), 257 (13)
Daidzin	6.120	37.1 ± 12.2	250	417.1178	MS^2^ (417):255 (100)	415.1042	—
Amoxicillin	2.690	4.9 ± 2.3	270	366.1084	MS^2^ (366):114 (100), 208 (17)	—	—
Glucuronide	2.547	4.3 ± 2.6	195	—	—	175.0420	MS^2^ (175):62 (100), 157 (23), 79 (22), 117 (19)

^a^Δ*P* was calculated using the following equation: Δ*P* = (*P*_e_ − *P*_c_)/*P*_e_ × 100, where *P*_e_ and *P*_c_ are the peak areas in the experiment and control, respectively. Data were obtained from 3 independent experiments and are expressed as mean ± SD.

**Table 2:** LC/MS data and assignment of 15 bioactive constituents in PR extract.

No.	*t*_R_ (min)	*∆P* ^b^ (%, *n* = 3)	UV *λ*max (nm)	[M+H]^+^ ([M+Na]^+^) *m*/*z*	ESI-MS^n^(+) *m*/*z* (abundance)	[M-H]^−^ *m*/*z*	ESI-MS^n^(-) *m*/*z* (abundance)	Predicted formula	Meas. (*m*/*z*)	Pred. (*m*/*z*)	Diff. (ppm)	DBE	Assigned identification
P1	31.593	81.43 ± 14.91	200	409.1899	MS^2^ (409):247 (100)	407.0915	MS^2^ (407):361 (100), 362 (23), 354 (16), 283 (16), 248 (15), 108 (15), 230 (14), 219 (14), 202 (14), 154 (12), 104 (11)	C_23_H_36_O_6_	408.1502	408.2597	-1.568	6	Unidentified
P2	44.173	93.26 ± 9.12	220, 255	367.1151	MS^2^ (367):209 (100), 210 (10)	365.2104	MS^2^ (365):329 (100), 191 (10), 298 (10)	C_18_H_6_O_9_	366.2184	366.0030	-1.73	16	(−)-*cis*-(3′R,4′R)-3′-angeloylkhellactone or (−)-*cis*-(3′R,4′R)-4′-angeloylkhellactone
P3	49.950	92.16 ± 5.36	325	367.1154	MS^2^ (367):267 (100), 203 (16) MS^2^ (345):203 (100), 204 (14), 187 (10) MS^3^ (203):203 (100), 175 (27), 204 (15)	343.9714	MS^2^ (343):99 (100), 261 (91), 203 (75), 175 (32), 262 (25), 280 (15), 243 (12), 229 (11) MS^3^ (261):203 (100), 175 (76), 69 (64), 145 (53), 189 (42), 223 (22), 161 (21), 119 (21), 89 (17), 195 (11), 118 (11), 186 (11)	C_12_H_8_O_12_	344.1154	344.0040	-2.99	9	Pd-C-I
P4	51.160	93.83 ± 12.52	200, 325	367.1143	MS^2^ (367):267 (100), 203 (23), 204 (15), 268 (14), 367 (12)	365.2106	—	C_18_H_6_O_9_	366.2145	366.0030	-1.73	16	(−)-*cis*-(3′R,4′R)-3′-angeloylkhellactone or (−)-*cis*-(3′R,4′R)-4′-angeloylkhellactone
P5	53.397	89.59 ± 16.01	325	367.1150	MS^2^ (367):267 (15)	343.1196	MS^2^ (343):215 (100), 99 (60), 95 (16), 69 (12), 172 (10), 151 (10)	C_12_H_8_O_12_	344.1270	344.0040	-2.99	9	(+)-*trans*-(3′S, 4′R)-3′-angeloylkhellactone
P6	58.633	73.81 ± 10.45	200, 325	376.1389	MS^2^ (376):245 (100), 227 (50), 287 (17), 246 (14) MS^2^ (245):203 (84), 175 (24), 145 (20), 246 (15), 204 (13)	351.2317	MS^2^ (351):59 (100), 277 (75), 351 (75), 352 (16), 278 (13)	C_17_H_20_O_8_	352.2133	352.1200	13.44	8	Unidentified
P7	60.897	65.58 ± 8.96	200, 255, 320	369.0947	MS^2^ (369):267 (100), 369 (30), 203 (22), 309 (19), 227 (10) MS^3^ (267):143 (100), 102 (82), 161 (60), 184 (53), 267 (43), 269 (36), 145 (21), 238 (13), 91 (10)	—	MS^2^ (345):299 (100), 277 (65), 300 (22), 69 (21), 95 (16), 278 (14)	C_18_H_18_O_7_	346.1107	346.0947	15.30	10	Qianhucoumarin D
^a^P8	63.003	73.82 ± 7.96	200, 325	409.1261	MS^2^ (409):245 (100), 227 (41) MS^3^ (245):189 (100), 144 (77), 180 (32), 175 (32), 163 (30), 187 (24), 161 (24), 186 (20), 83 (17), 198 (11)	385.3148	—	C_21_H_22_O_7_	386.3178	386.1410	10.35	11	Praeruptorin A
P9	63.300	72.95 ± 8.36	200, 325	409.1261	MS^2^ (409):245 (100), 309 (93), 227 (35), 409 (30), 83 (25), 310 (21), 246 (17) MS^3^ (245):175 (48), 189 (16), 246 (16), 217 (15), 187 (14)	385.3148	—	C_21_H_22_O_7_	386.3178	386.1410	10.35	11	Unidentified
P10	68.660	71.51 ± 9.92	200, 325	411.1402	MS^2^ (411):217 (100), 175 (83), 187 (71), 92 (56), 201 (51), 83 (42), 69 (42), 53 (36)	387.1149	—	C_21_H_24_O_7_	388.1180	388.1570	-4.26	10	Peucedanocoumarin I
P11	69.150	84.02 ± 8.36	205	449.1563	MS^2^ (449):245 (100), 227 (71), 246 (17), 175 (14), 228 (13)	425.3047	—	C_22_H_18_O_9_	426.2538	426.0990	12.83	14	Unidentified
^a^P12	69.713	71.44 ± 9.38	200	449.1562	MS^2^ (449):83 (100), 227 (21), 349 (13)	425.3046		C_24_H_26_O_7_	426.2535	426.0990	12.83	14	Praeruptorin B
^a^P13	70.043	86.51 ± 9.38	200	449.1658	MS^2^ (449):83 (100), 227 (30), 55 (17), 349 (17), 327 (13)	425.3046	—	C_24_H_26_O_7_	426.2538	426.0990	12.83	14	Praeruptorin D
P14	71.390	83.44 ± 10.32	200	449.1572	MS^2^ (449):409 (100)	425.3050		C_24_H_26_O_7_	426.2535	426.0990	12.83	14	Unidentified
^a^P15	71.927	89.21 ± 13.59	200	451.1780	MS^2^ (451):227 (37), 327 (17), 349 (15)	427.2556	—	C_24_H_28_O_7_	428.3917	428.1890	-6.90	11	Praeruptorin E

^a^Compared with standards. ^b^Δ*P* was calculated using the following equation: Δ*P* = (*P*_e_–*P*_c_)/*P*_e_ × 100, where *P*_e_ and *P*_c_ are the peak areas in the experiment and control, respectively. Data were obtained from 3 independent experiments and are expressed as mean ± SD.

## Data Availability

The data used to support the findings of this study are available from the corresponding author upon request.

## Supplementary Materials

Supplementary Materials Figure S1: changes in ∆Ψm of HM after persistent heating in boiling water for 2 h. Figure S2: observation of isolated HM using an optical microscope. Figure S3: effect of mitochondrial concentration on screening of bioactive constituents from the PR extract. Figure S4: effect of concentration of the PR sample on screening of HM-targeting compounds. Figure S5: effect of incubation time on screening of bioactive compounds from the PR extract. Table S1: HPLC–DAD conditions for all analyzed samples.
