# Supplementary material for: Efficiently Capturing Mitochondria-Targeted Constituents with Hepatoprotective Activity from Medicinal Herbs
Source: Oxid Med Cell Longev. 2019 Apr 9;2019:4353791. doi: 10.1155/2019/4353791 (PMC6481013; doi:10.1155/2019/4353791)
Supplement: Supplementary Materials — Figure S1: changes in ∆Ψm of HM after persistent heating in boiling water for 2 h. Figure S2: observation of isolated HM using an optical microscope. Figure S3: effect of mitochondrial concentration on screening of bioactive constituents from the PR extract. Figure S4: effect of concentration of the PR sample on screening of HM-targeting compounds. Figure S5: effect of incubation time on screening of bioactive compounds from the PR extract. Table S1: HPLC–DAD conditions for all analyzed samples. [file 4353791.f1.pdf]

SUPPLEMENTARY INFORMATION

Efficiently-Capturing Mitochondria-Targeted Constituents with Hepatoprotective Activity from Medicinal Herbs

Xing-XinYang [a,b,#], Li Liang [a,b,#], Xin Liu [c], Feng-Jiao Li [a,b], Jin-Cai Dong [a,b], Jian-Kang Mu [a,b], Lin-Xi Zeng [a,b], Qian Bi [a,b], Wen Gu [a,b], Jie Yu [a,b,]*

[a] College of Pharmaceutical Science, Yunnan University of Traditional Chinese Medicine, 1076 Yuhua Road, Kunming 650500, Yunnan Province, P.R. China

[b] Kunming Key Laboratory for Metabolic Diseases Prevention and Treatment by Chinese Medicine, 1076 Yuhua Road, Kunming 650500, Yunnan Province, P.R. China

[c] Beijing Entry-Exit Inspection and Quarantine Bureau, 6 Tianshuiyuan Street, Beijing 100026, China

[#] Both authors contributed equally to this work

Corresponding author:

Dr. Jie Yu (Tel./Fax.: +86-871-65933303; Email: cz.yujie@gmail.com)

Table of contents

Supplementary Experimental Procedures

Supplementary Figures and Tables

1. **FIGURE S1.** Changes in $\Delta\Psi m$ of HM after persistent heating in boiling water for 2 h.
2. **FIGURE S2.** Observation of isolated HM using an optical microscope.
3. **FIGURE S3.** Effect of mitochondrial concentration on screening of bioactive constituents from PR extract.
4. **FIGURE S4.** Effect of concentration of PR sample on screening of HM-targeting compounds.
5. **FIGURE S5.** Effect of incubation time on screening of bioactive compounds from PR extract.
6. **Table S1.** HPLC–DAD conditions for all analyzed samples.

Supplementary Experimental Procedures

Preparation of freeze-dried powder of Peucedani Radix (PR) extract. Pulverized dried samples of PR (50 g) were soaked for 30 min in 500 mL methanol, and extracted using ultrasonication (500 W, 40 KHz) for 60 min. The extracted solution was filtered and collected, and the residue was ultrasonicated (500 W, 40 KHz) with 400 mL of 70% methanol for another 60 min. Extracted solutions were then filtered. Next, the two successive filtrates were mixed and evaporated using an N-1100D-WD rotatory evaporator (Ai Lang Instrument Co., Ltd., Shanghai, China) at 45 ˚C under reduced pressure. Finally, the concentrate was lyophilized to powder with a FD8-10B freeze dryer (SIM International Group Co. Ltd., Newark, DE, USA). Obtained powder of PR extracts was stored in a dryer at room temperature until use.

Janus green B staining. Janus green B (Beijing Solarbio Science & Technology Co., Ltd. Beijing, China), a dye specific for the mitochondrial membrane [1], was used for the initial observation of isolated hepatic mitochondria (HM). HM pellets were resuspended in 200 μL Reagent E from the Mitochondria Isolation Kit (Genmed Scientifics Inc., Arlington, MA, USA), and mixed using 100 μL mixed dye solution containing 0.3% (w/v) Janus green B and 0.15% (w/v) neutral red. The mixture was incubated for 10 min at room temperature and HM was observed using a Leica DMILLED Inverted Fluorescence Microscope (Leica Microsystems GmbH, Wetzlar, Germany).

Transmission electron microscopy. Transmission electron microscopy was applied for verifying the integrity and purity of prepared HM. Freshly prepared HM pellets were fixed overnight using 2.5% glutaraldehyde in phosphate buffer (0.01 M, pH 7.4)

and post-fixed by 1% osmium tetroxide in phosphate buffer (0.24 M, pH 7.4). Then, samples were washed with PBS buffer, dehydrated with ethanol and a graded acetone series (20%, 30%, 50%, 70%, 90%, and 100%) and embedded in Epon resin. Thin sections were cut by a Leica UC6 microtome and sequentially double-stained with uranyl acetate and lead citrate before electron microscopy. Images were obtained from a JEM-100cx transmission electron microscope (JEOL Ltd., Tokyo, Japan).

Measurement of mitochondrial membrane potential ($\Delta\Psi m$). Determination of $\Delta\Psi m$ was performed using rhodamine 123 (Rh123) fluorescence quenching [2]. At a high $\Delta\Psi m$ level, most of the Rh123 is accumulated in the mitochondrial matrix and quenches. However, at lower $\Delta\Psi m$ levels, Rh123 is released, resulting in dequenching and an enhancement in Rh123 fluorescence. In brief, isolated HM were resuspended in a buffer (0.25 M sucrose, 5 mM $MgCl_2$, 10 mM KCl, 5 mM KH_2PO_4, 10 mM HEPES, 10 mM succinate, pH 7.4) to a final concentration of 1 g/L, and incubated with 5 μL Rh123 (1g/L) for 30 min at 37 ˚C. The $\Delta\Psi m$-dependent quenching of Rh123 fluorescence was tested with an Infinite M200 Pro Multi-functional Microplate Reader (Dongsheng Innovation Biotechnology Co., Ltd., Beijing, China) set at 484 nm excitation and 534 nm emission wavelengths. Subsequently, tested HM samples were treated with 1 μL CCCP (10 mM, Beyotime Institute of Biotechnology, Haimen, China) for 20 min at room temperature, and fluorescence was measured using conditions mentioned before.

***In vivo* hepatoprotective activity assay.** 1) Assessment of serum marker enzymes including alanine transaminase (ALT) and aspartate transaminase (AST): after blood collection, serum was separated by centrifugation at 3000 × *g* for 10 min at 4˚C.

Serum analysis of various liver marker enzymes, including ALT and AST were determined using the corresponding diagnostic kits (Nanjing Built Biological Engineering Research Institute, Nanjing, China) in accordance with the manufacturer's instructions in a Roche Cobas 8000 Automatic Multifunction-Biochemical Analyzer (Roche Diagnostics, Mannheim, Germany).

2) Histopathological examination of liver tissues: liver tissue from a portion of the left lobe was removed, fixed in 10% buffered formalin solution (pH 7.0) for at least 24 h, then dehydrated and embedded in paraffin. After hematoxylin and eosin (H&E) staining, the histopathological characters, including fatty changes, necrosis, ballooning degeneration and cell infiltration, were observed under a light microscope (Nikon Eclipse TE 2000-U, Nikon, Tokyo, Japan) and photographed at 100 × magnification.

3) Assessment of antioxidant indexes in liver tissues including malondialdehyde (MDA), superoxide dismutase (SOD), and glutathione (GSH): liver tissue (~200 mg) was weighed and homogenized in 1.8 mL ice-cold physiological saline, then centrifuged at 3000 × *g* for 10 min at 4˚C. Supernatants were collected and used to determine the levels of SOD, MDA, GSH and total protein concentrations using the commercially available diagnostic kits in accordance with the manufacturer's instruction (Nanjing Built Biological Engineering Research Institute, Nanjing, China) in a SpectraMax Plus 384 Microplate Reader (Molecular Devices, Sunnyvale, CA, USA). The levels of SOD, MDA and GSH were normalized with the total protein contents.

4) Assessment of mitochondria-involved indexes (MDA, ATPase, $\Delta\Psi m$, and mPTP): HM isolated from liver tissue (200 mg) were resuspended in 100 μL of ice-cold physiological saline, and used to determination levels of MDA and ATPase by

commercially available diagnostic kits according to the manufacturer's instructions (Nanjing Built Biological Engineering Research Institute, Nanjing, China) in a SpectraMax Plus 384 Microplate Reader (Molecular Devices, Sunnyvale, CA, USA). Isolated HM were resuspended in swelling buffer (120 mM KCl, 20 mM MOPS, 10 mM Tris-HCl, and 5 mM KH_2PO_4, pH 7.4) to a final concentration of 0.25 g/L, and 250 μM $CaCl_2$ was added for 15 min to induce opening of mPTP. After that, the A_{520} was determined in triplicate using a UV–VIS spectrophotometer (AOE instrument (Shanghai) Co., Ltd., Shanghai, China). Moreover, HM were resuspended in measurement medium (0.25 M sucrose, 5 mM $MgCl_2$, 10 mM KCl, 5 mM KH_2PO_4, 10 mM HEPES, and 10 mM succinate, pH 7.4) to a final concentration of 1 g/L and were then incubated with Rh123 (5 μL; 1 g/L) for 30 min at 37 ℃. The $\Delta\Psi$m-dependent quenching of Rh123 fluorescence was tested with an Infinite M200 Pro Multi-functional Microplate Reader (Dongsheng Innovation Biotechnology Co., Ltd., Beijing, China) that was set at 484 nm excitation and 534 nm emission wavelengths.

References

[1] S. Chaiyarit, and V. Thongboonkerd, "Comparative analyses of cell disruption methods for mitochondrial isolation in high-throughput proteomics study," *Analytical Biochemistry*, vol. 394, no. 2, pp. 249–258, 2009.

[2] N. Zamzami, D. Métivier, and G. Kroemer, "Quantitation of mitochondrial transmembrane potential in cells and in isolated mitochondria," *Methods in Enzymology*," vol. 322, pp. 208–213, 2000.

Supplementary Figures and Tables

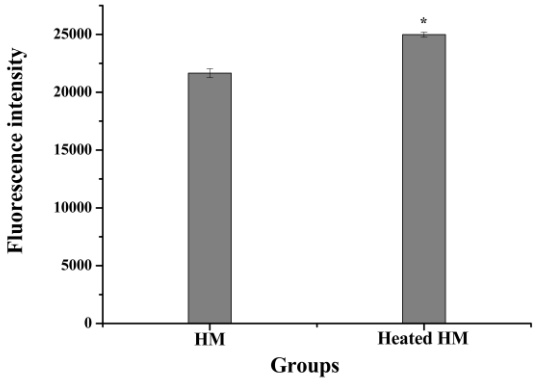

FIGURE S1. Changes in ΔΨm of HM after persistent heating in boiling water for 2 h. The significant increase of Rh 123 fluorescence in heated HM indicated the ΔΨm collapse, suggesting the loss of their primary biological function. ΔΨm was examined as the difference in Rh 123 uptake by normal and heated HM and expressed in fluorescence intensity units. Values are expressed as the mean of three individual experiments (S.D.). Statistical significance between normal and heated HM is indicated by * ($P < 0.05$).

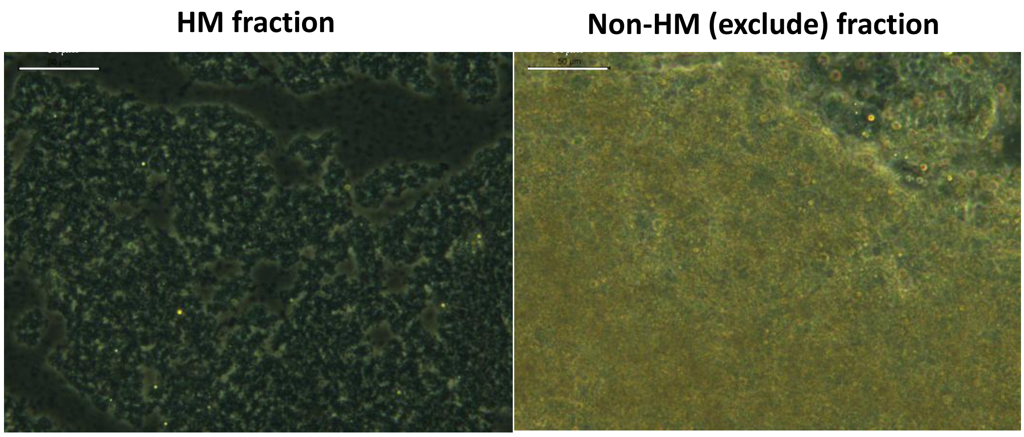

FIGURE S2. Observation of isolated HM using an optical microscope. Janus green B, which specifically stains the mitochondrial membrane, was applied together with neutral red (which stains lysosomes and can also stain the Golgi apparatus) for initial observation of the isolated HM. In all panels, the original magnification was 400 ×. The experimental procedure of Janus green B staining is presented in the Supplementary Information.

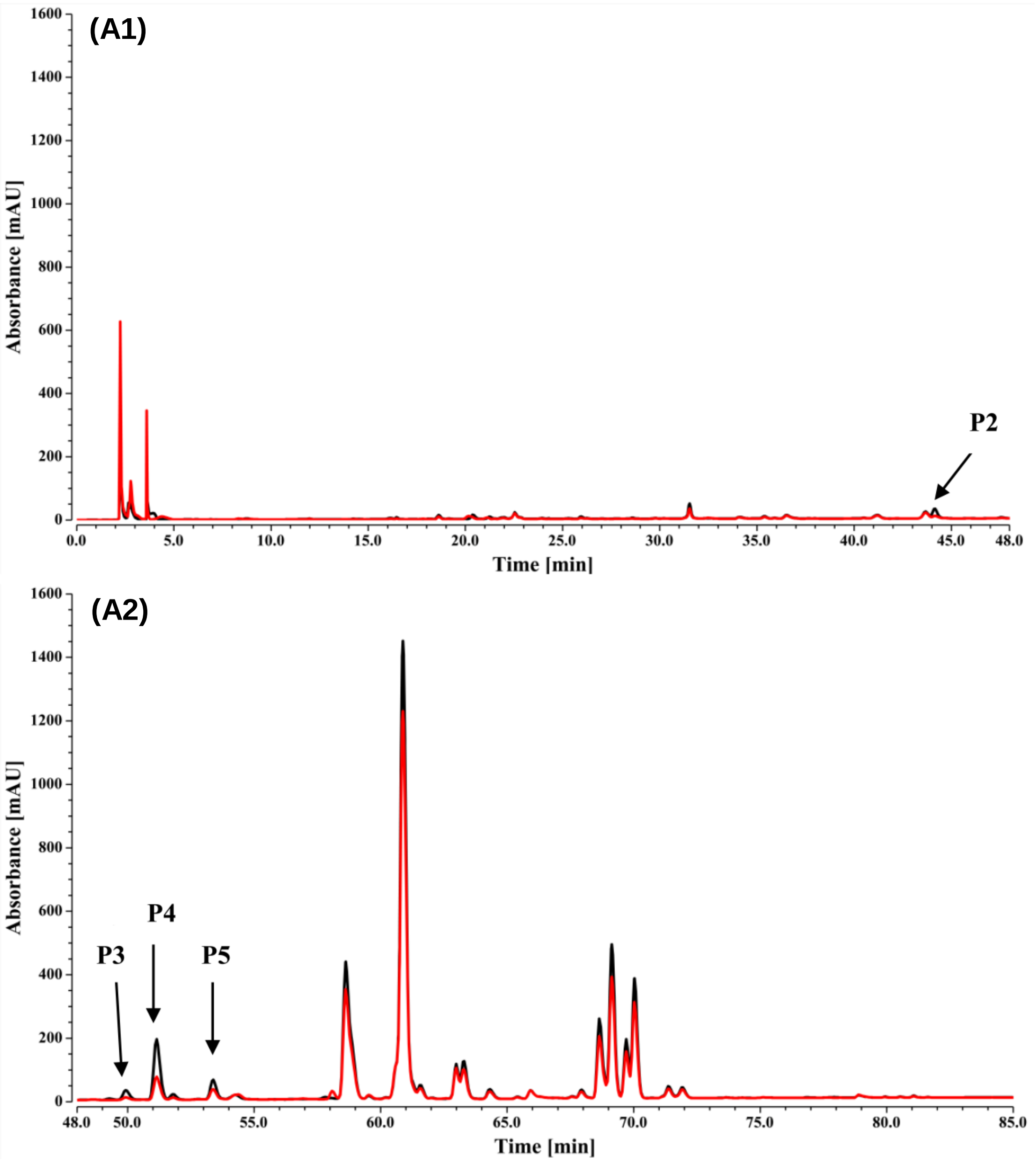
(A1)
Absorbance [mAU]
Time [min]
P2
(A2)
Absorbance [mAU]
Time [min]
P3
P4
P5

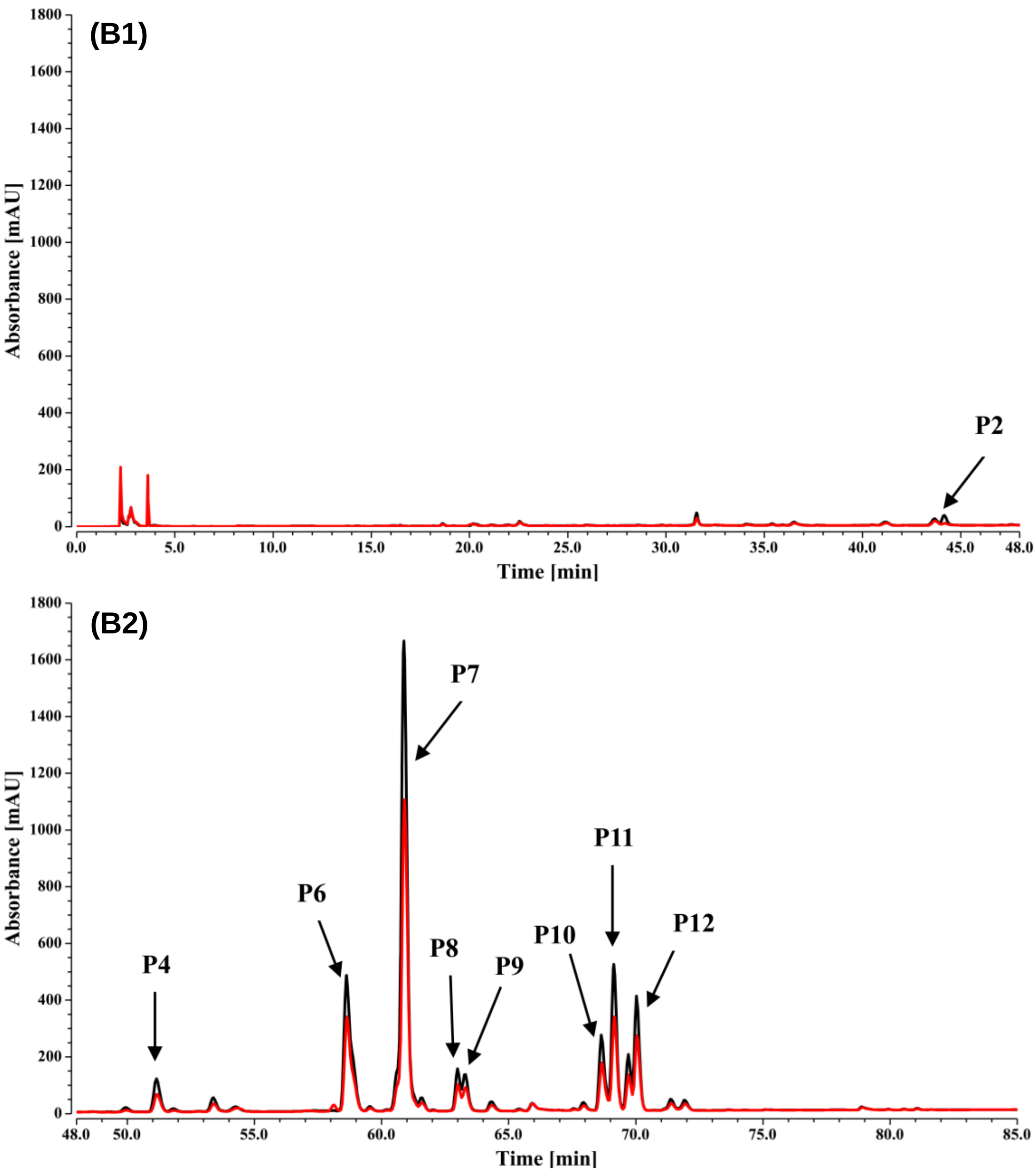
(B1)
Absorbance [mAU]
Time [min]
P2
(B2)
Absorbance [mAU]
Time [min]
P4
P6
P7
P8
P9
P10
P11
P12

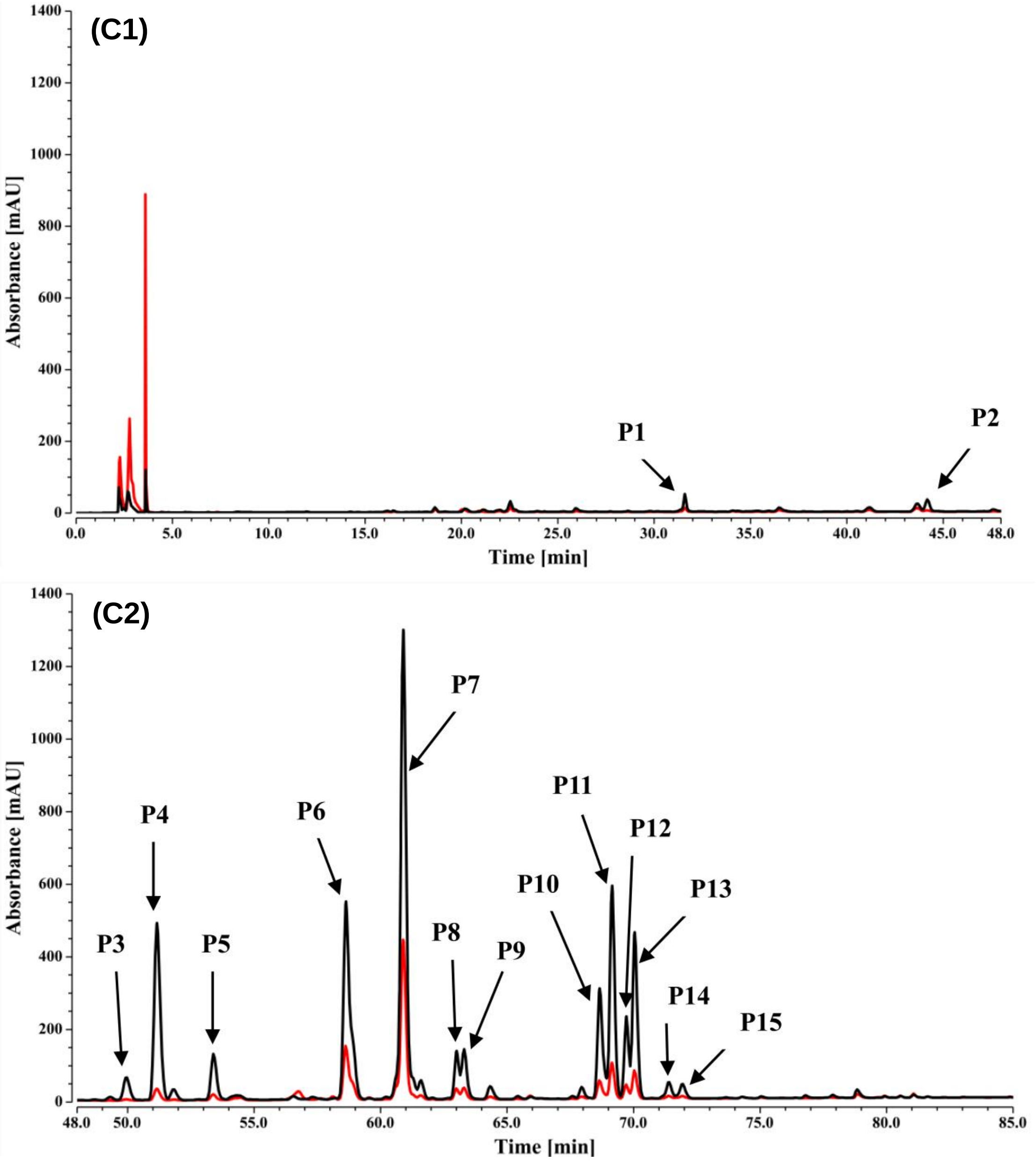

FIGURE S3. Effect of mitochondrial concentration on screening of active constituents from PR extract. The concentrations of mitochondria were 0.25 (A1 and A2; A1, 0–48 min; A2, 48–85 min), 0.50 (B1 and B2; B1, 0–48 min; B2, 48–85 min) and 1.00 g/L (C1 and C2; C1, 0–48 min; C2, 48–85 min). Compared with thecontrol, i.e., denatured HM (red line), HPLC chromatograms of searched PR extract exhibited fifteen peaks (P1–P15) that were promoted due to specific binding with HM (black line). The concentration of PR sample and incubation time were 12.38 g/L and 90 min, respectively.

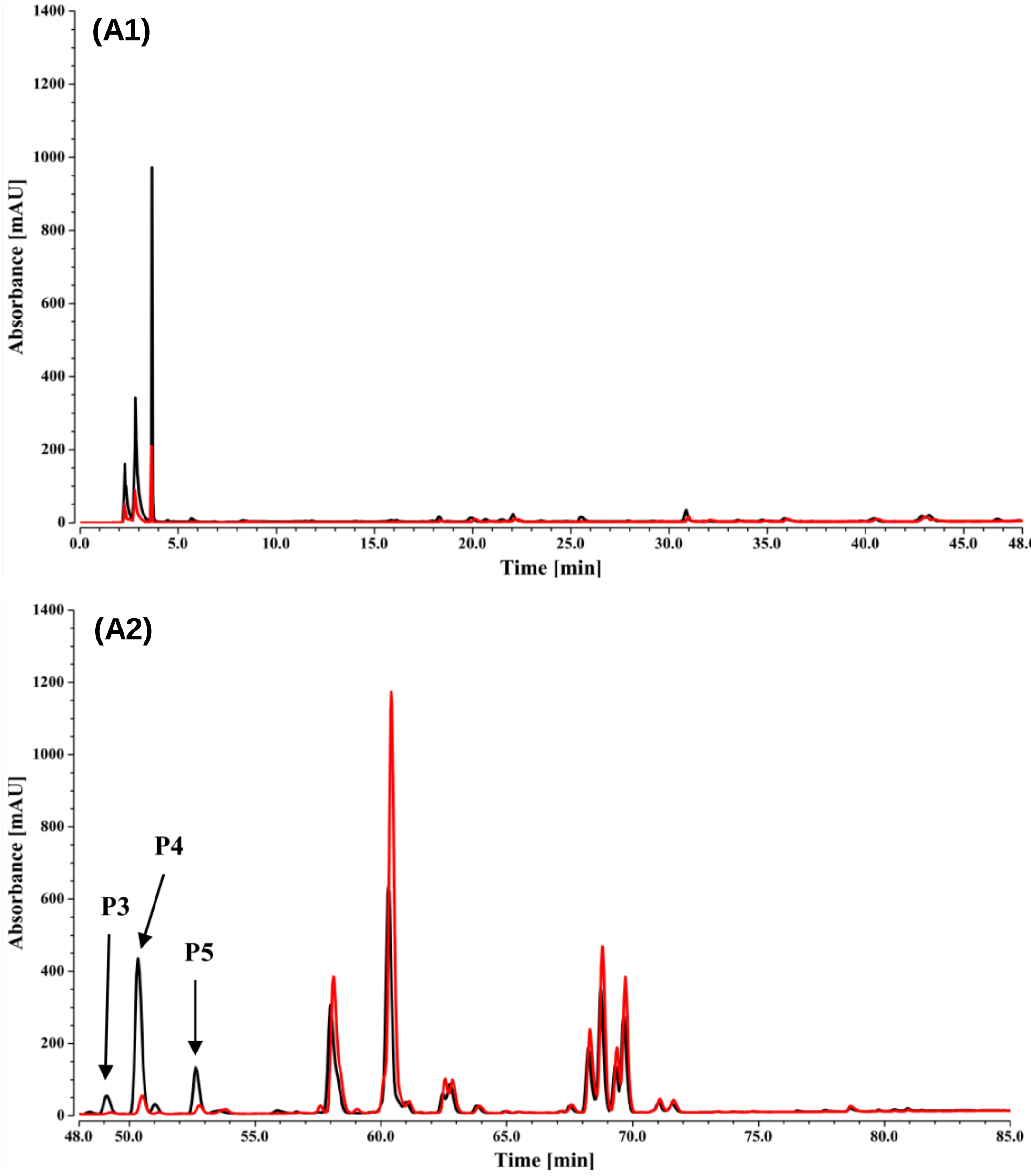
(A1)
Absorbance [mAU]
Time [min]
(A2)
P3
P4
P5
Absorbance [mAU]
Time [min]

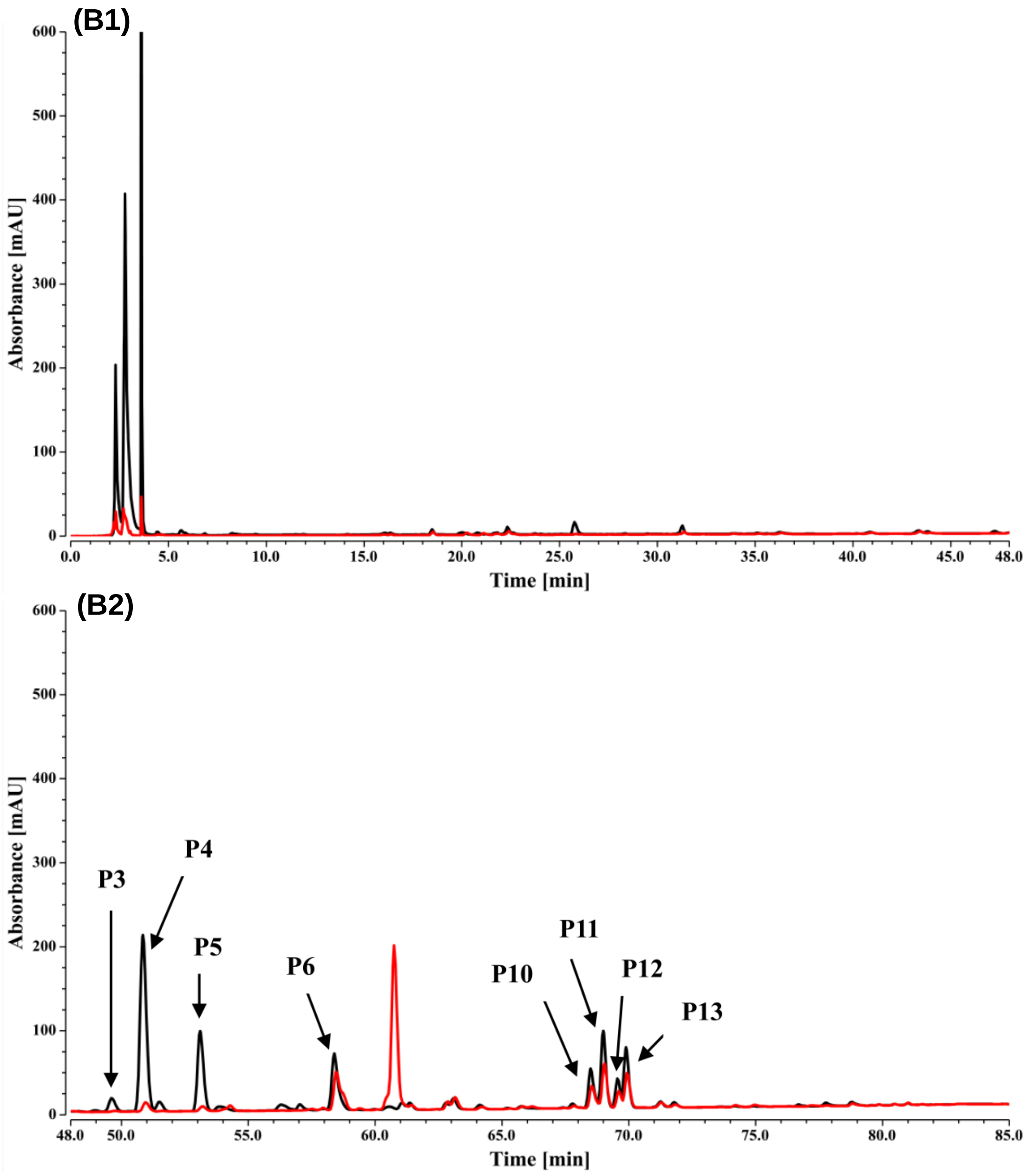
(B1)
Absorbance [mAU]
Time [min]
(B2)
Absorbance [mAU]
Time [min]
P3
P4
P5
P6
P10
P11
P12
P13

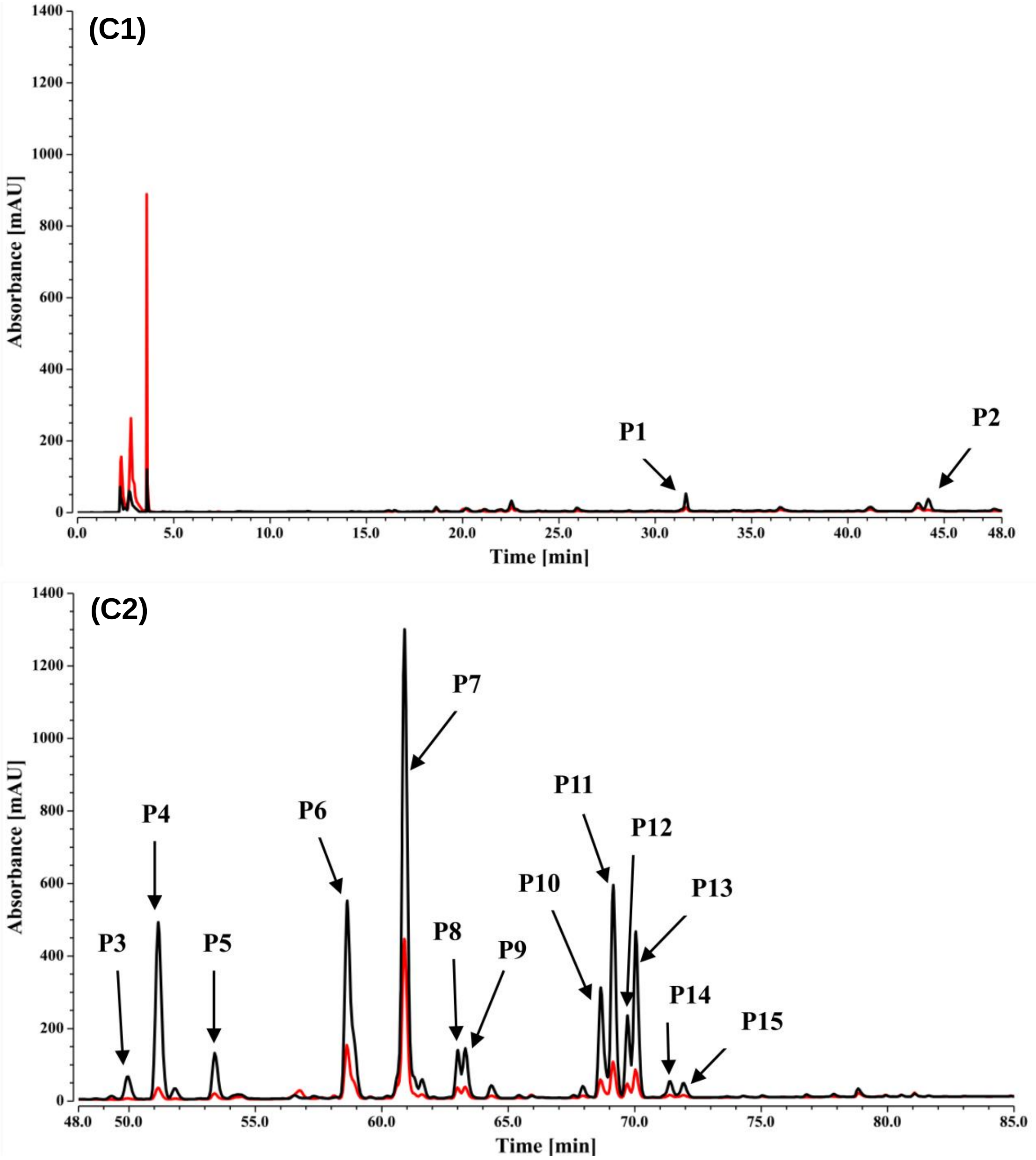

FIGURE S4. Effect of concentration of PR sample on screening of HM-targeting compounds. The concentrations of PR sample were 3.10 (A1 and A2; A1, 0–48 min; A2, 48–85 min), 6.19 (B1 and B2; B1, 0–48 min; B2, 48–85 min) and 12.38 g/L (C1 and C2; C1, 0–48 min; C2, 48–85 min). HPLC chromatograms of PR sample are displayed for ultrafiltrates obtained with active HM (black line) and denatured HM (red line) as the control. Fifteen peaks (P1–P15) showed significant enhancement of the peak area when compared with the control. The concentration of HM and incubation time were 1.00 g/L and 90 min, respectively.

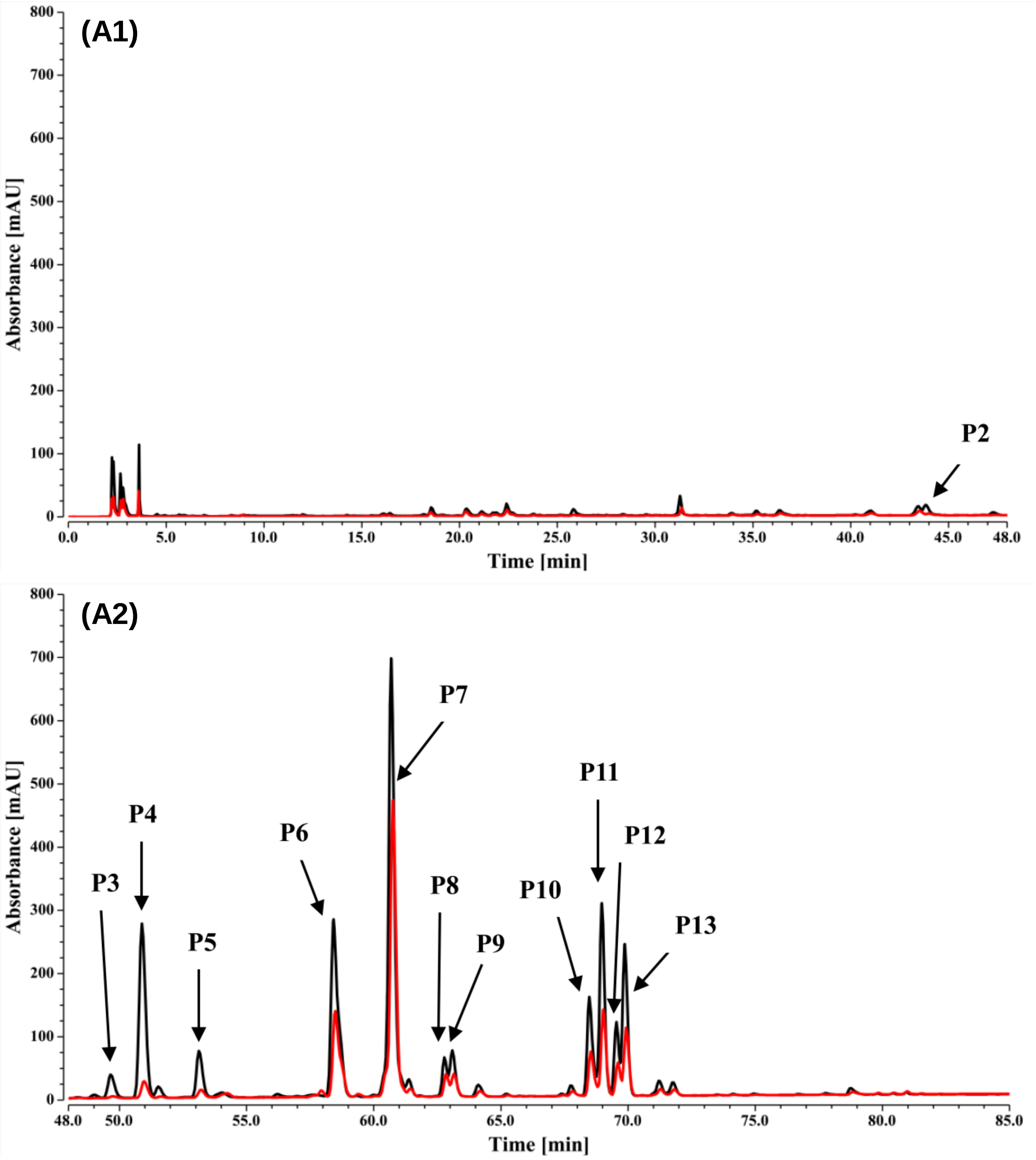
(A1)
Absorbance [mAU]
Time [min]
P2
0
100
200
300
400
500
600
700
800
0.0
5.0
10.0
15.0
20.0
25.0
30.0
35.0
40.0
45.0
48.0
(A2)
Absorbance [mAU]
Time [min]
P3
P4
P5
P6
P7
P8
P9
P10
P11
P12
P13
0
100
200
300
400
500
600
700
800
48.0
50.0
55.0
60.0
65.0
70.0
75.0
80.0
85.0

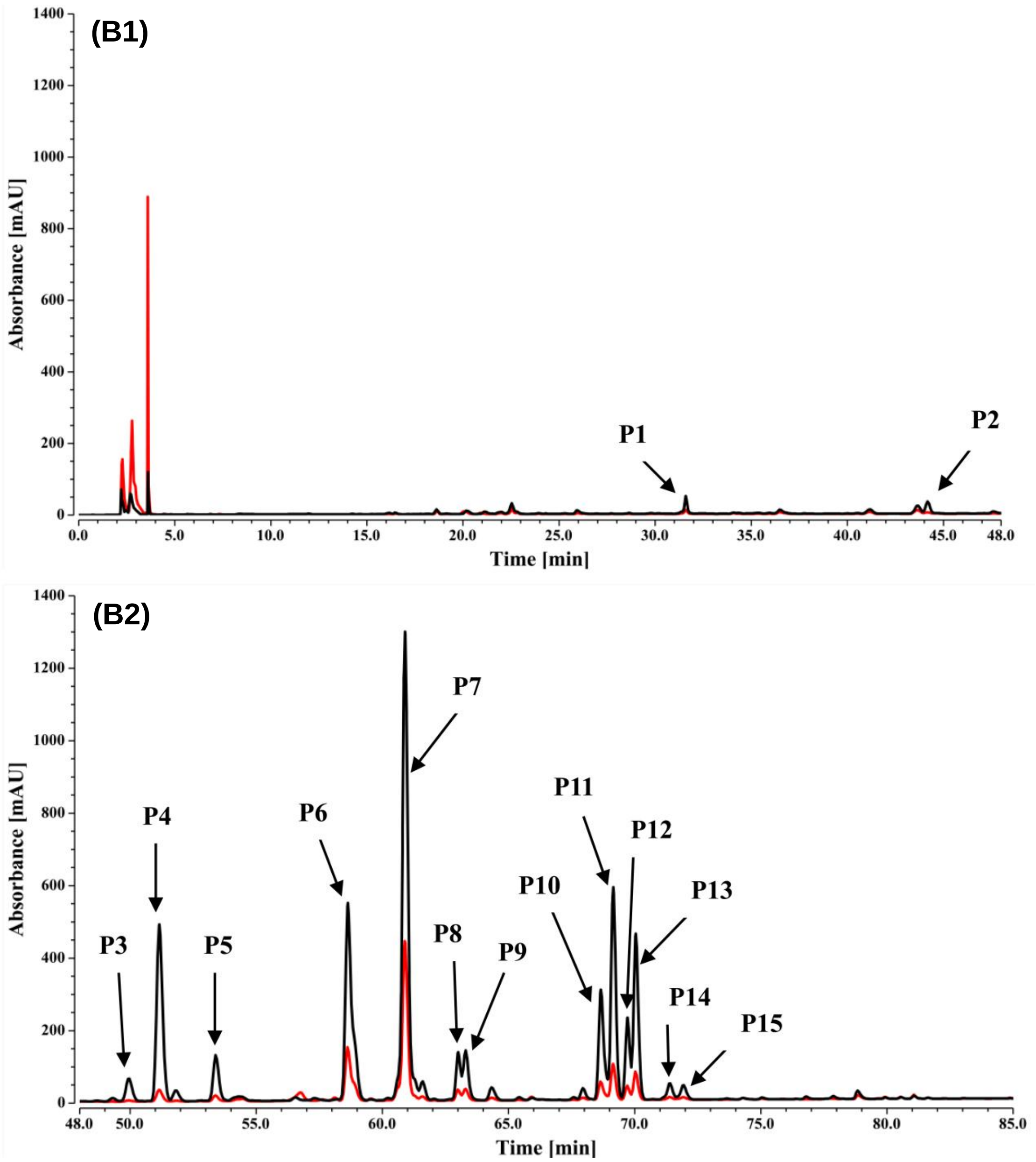
(B1)
Absorbance [mAU]
Time [min]
P1
P2
(B2)
Absorbance [mAU]
Time [min]
P3
P4
P5
P6
P7
P8
P9
P10
P11
P12
P13
P14
P15

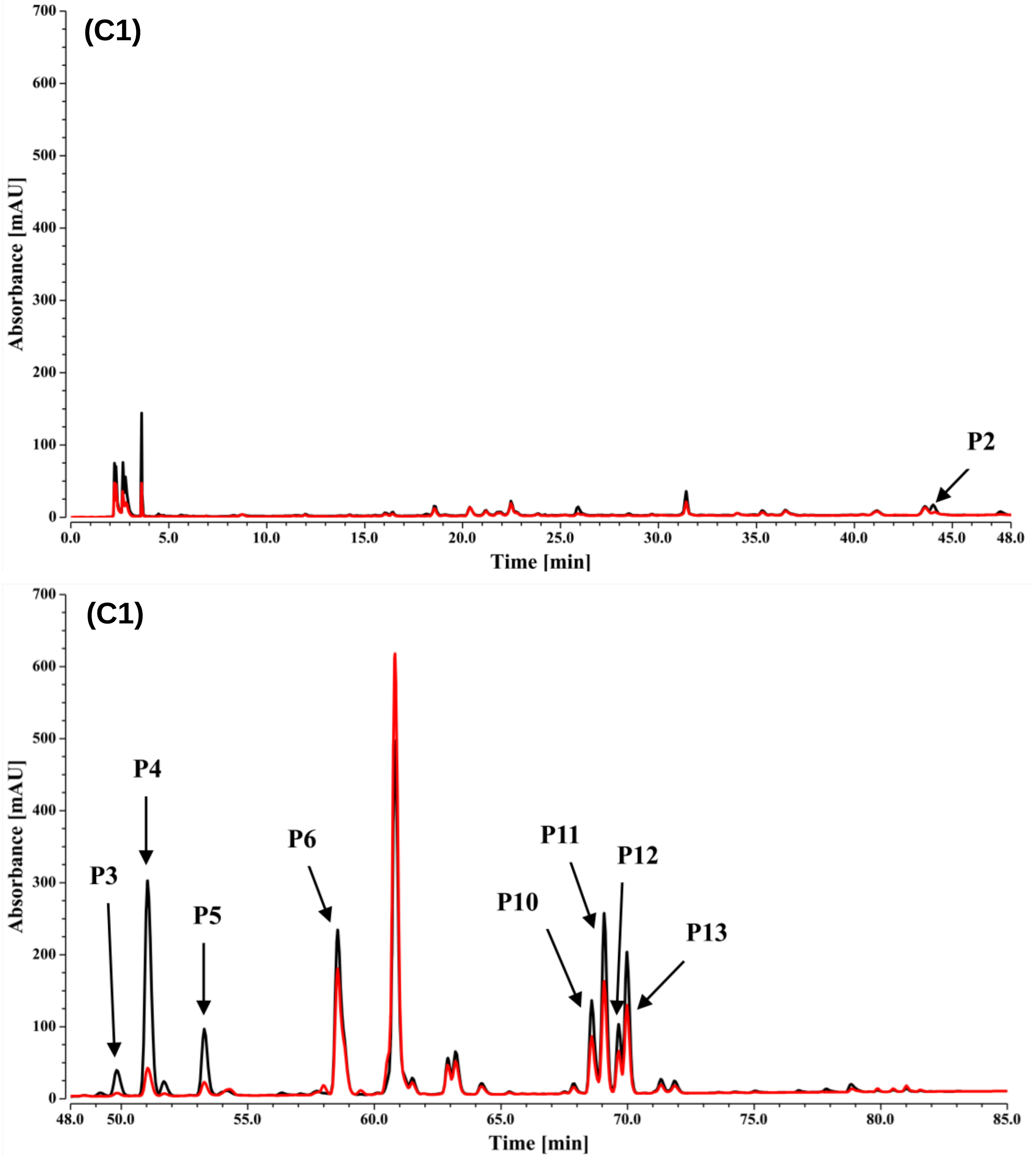

FIGURE S5. Effect of incubation time on screening of bioactive constituents from PR extract. Incubation times were 60 (A1 and A2; A1, 0–48 min; A2, 48–85 min), 90 (B1 and B2; B1, 0–48 min; B2, 48–85 min) and 120 min (C1 and C2; C1, 0–48 min; C2, 48–85 min). Compared to the control, which was comprised of denatured HM (red line), HPLC chromatograms of screened PR extract showed fifteen peaks (P1–P15) that were enhanced because of specific binding to HM (black line). The concentrations of HM and PR sample were 1.00 g/L and 12.38 g/L, respectively.

Table S1. HPLC–DAD conditions for all analyzed samples

**Sample**	**Chromatographic column**	**Mobile phase**	**Eluent gradient**	**Injection volume (μL)**	**Column temperature (℃)**	**Flow rate (mL/min)**	**Detection wavelength (nm)**
Silybin	Agilent Zorbax SB-C18 column (250mm×4.6mm I.D., 5μm)	Water-formic acid (100:0.1, v/v) and methanol (25:75 v/v)	Isocratic elution	10	30	1.0	288
Daidzin	Agilent Zorbax SB-C18 column (250mm×4.6mm I.D., 5μm)	Water-acetic acid (100:0.1, v/v) and methanol (60:40 v/v)	Isocratic elution	10	30	1.0	250
Amoxicillin	Agilent Zorbax SB-C18 column (250mm×4.6mm I.D., 5μm)	Water- formic acid (100:0.1, v/v) and methanol (85:15 v/v)	Isocratic elution	10	30	1.0	228
Glucuronolode	Agilent Zorbax SB-C18 column (250mm×4.6mm I.D., 5μm)	Water and methanol (35:65 v/v)	Isocratic elution	10	40	1.0	221
Mixed standard solution (comprised of silybin, daidzin, amoxicillin and glucuronolode)	Agilent Zorbax SB-C18 column (250mm×4.6mm I.D., 5μm)	Water (A) and methanol (B)	0 min, 2%B; 3 min, 2%B; 6 min, 95%B; 10 min, 100%B	10	30	1.0	221
PR extract	Agilent Zorbax SB-C18 column (250mm×4.6mm I.D., 5μm)	Water (A) and acetonitrile (B)	0 min, 5%B; 10 min, 15%B; 20 min, 25%B; 30 min, 35%B; 45 min, 45%B; 60 min, 65%B; 70 min, 80%B; 80 min, 95%B; 85 min, 100%B	20	30	1.0	203
